# PD-L1 signaling on human memory CD4+ T cells induces a regulatory phenotype

**DOI:** 10.1371/journal.pbio.3001199

**Published:** 2021-04-26

**Authors:** Giorgia Fanelli, Marco Romano, Estefania Nova-Lamperti, Mariana Werner Sunderland, Alessandra Nerviani, Cristiano Scottà, Michele Bombardieri, Sergio A. Quezada, Steven H. Sacks, Randolph J. Noelle, Costantino Pitzalis, Robert I. Lechler, Giovanna Lombardi, Pablo D. Becker

**Affiliations:** 1 MRC Centre for Transplantation, School of Immunology and Microbial Sciences, King’s College London, Guy’s Hospital, London, United Kingdom; 2 Cancer Immunology Unit, University College London (UCL) Cancer Institute, London, United Kingdom; 3 Centre for Experimental Medicine and Rheumatology, William Harvey Research Institute, Queen Mary University of London and Barts’ Health NHS Trust, London, United Kingdom; 4 Department of Microbiology and Immunology, Norris Cotton Cancer Center Geisel School of Medicine at Dartmouth, Lebanon, New Hampshire, United States of America; 5 King’s Health Partners, London, United Kingdom; Children’s Hospital of Philadelphia and The University of Pennsylvania School of Medicine, UNITED STATES

## Abstract

Programmed cell death protein 1 (PD-1) is expressed on T cells upon T cell receptor (TCR) stimulation. PD-1 ligand 1 (PD-L1) is expressed in most tumor environments, and its binding to PD-1 on T cells drives them to apoptosis or into a regulatory phenotype. The fact that PD-L1 itself is also expressed on T cells upon activation has been largely neglected. Here, we demonstrate that PD-L1 ligation on human CD25-depleted CD4^+^ T cells, combined with CD3/TCR stimulation, induces their conversion into highly suppressive T cells. Furthermore, this effect was most prominent in memory (CD45RA^−^CD45RO^+^) T cells. PD-L1 engagement on T cells resulted in reduced ERK phosphorylation and decreased AKT/mTOR/S6 signaling. Importantly, T cells from rheumatoid arthritis patients exhibited high basal levels of phosphorylated ERK and following PD-L1 cross-linking both ERK signaling and the AKT/mTOR/S6 pathway failed to be down modulated, making them refractory to the acquisition of a regulatory phenotype. Altogether, our results suggest that PD-L1 signaling on memory T cells could play an important role in resolving inflammatory responses; maintaining a tolerogenic environment and its failure could contribute to ongoing autoimmunity.

## Abstract 2

This study shows that programmed death cell receptor ligand 1 (PD-L1) signaling in memory CD4+ T cells from healthy individuals induces a regulatory phenotype; this mechanism seems to be defective in equivalent T cells from rheumatoid arthritis patients and could be in part responsible for the pathology.

## Introduction

Therapies for autoimmune diseases and chronic inflammation are mainly directed toward reestablishing a balance in the immune system. When the immune system responds to infectious agents, to tumor transformation, or to a vaccine antigen, several mechanisms exist to limit collateral damage leading to T cell–dependent immunopathology [1,2] However, not all the mechanisms intended to limit pathological immune responses and their specific molecular pathways are fully understood. Regulatory T cells (Tregs) are key players in maintaining immune homeostasis and tolerance to self [3]. FOXP3 is considered the master regulator of Tregs by controlling their development and function, although its sole expression is not sufficient to define a human Treg. Stimulation of effector CD4^+^ T cells by T cell receptor (TCR) and CD28 cross-linking results in a vigorous proliferation and transient up-regulation of FOXP3 [4,5] and, although these CD4^+^FOXP3^+^ T cells are suppressive, they rapidly lose FOXP3 expression and suppressive capacity [6]. Other molecules central to tolerance are the cytotoxic T-lymphocyte–associated antigen 4 (CTLA-4) and Programmed cell death protein 1 (PD-1). CTLA-4 exerts its inhibitory function by competing with CD28 for costimulatory ligands CD80 and CD86 or by stripping them from antigen-presenting cells (APCs), thereby reducing the costimulatory signals that T cells receive [7]. PD-1 can block a TCR-induced stop signal that limits T cell activation [8], as one of the mechanisms described for CTLA-4 [9], and favors inducible Treg (iTreg) generation from naïve T cells [10]. Furthermore, PD-1 ligand 1 molecule (PD-L1, also known as B7-H1 or CD274) has been described on APCs and tumor cells to induce cell death and promote iTreg cell conversion through the binding of PD-1 expressed on activated CD4^+^ T cells [10,11]. While CTLA-4 plays an important role in secondary lymphoid organs during the initial stage of T cell activation, PD-1 regulates activated T cells at a later stage, typically in peripheral tissues where PD-L1 is expressed under certain physiological and pathological conditions by hematopoietic (e.g., dendritic cells in the gut) and nonhematopoietic cells (e.g., tumor cells) [12,13]. PD-L1 binding to PD-1 on T cells triggers a signaling cascade that has been extensively investigated [14]. In contrast, a signaling cascade downstream of PD-L1 has been so far neglected due to its short cytoplasmic tail and the lack of obvious signal transduction motifs in its sequence. However, an early report showed that cancer cell resistance to apoptosis is lost following transduction with a PD-L1 construct lacking the cytoplasmic domain [15]. Recently, it has been shown that PD-L1 intracytoplasmic tail can trigger a signal cascade that make cancer cells resistant to interferon (IFN)-mediated cytotoxicity through a STAT3/caspase-7-dependent pathway [16]. By serendipity, Amarnath and colleagues observed changes in the percentage of T-bet, FOXP3, and CD80 on PD-L1-expressing T cells that was not further investigated [11] but was indicative of a PD-L1 reverse signaling. In addition, Diskin and colleagues recently showed that binding to PD-L1 induced STAT3-dependent “back signaling” in CD4 T cells that prevented activation and polarization [17].

In this study, we demonstrated that cross-linking of PD-L1 on CD4^+^CD25^−^ T cells, in combination with CD3/TCR, induced their conversion into highly suppressive iTregs from the memory pool by modulating the mitogen-activated protein kinase (MAPK) pathway, increasing STAT3 and STAT5 phosphorylation and decreasing ERK phosphorylation downstream to the TCR signaling pathway and antagonizing the AKT/mTOR pathway. Importantly, we showed that T cells from rheumatoid arthritis patients failed to modulate both ERK signaling and the AKT/mTOR pathway upon PD-L1 engagement. This could contribute to the defective resolution of inflammation leading to disease persistence and progression.

## Results

### Concomitant PD-L1 and TCR complex engagement activates CD4^+^CD25^−^ T cells

PD-1/PD-L1 signaling has been described as an inhibitory pathway that typically inhibits T cell responses following the binding of PD-1 expressed by T cells to its ligand PD-L1 expressed on B cells, macrophages, and dendritic cells. However, the fact that PD-L1 itself can be highly expressed on activated human CD4^+^ T cells has been overlooked.

**Fig 1:**
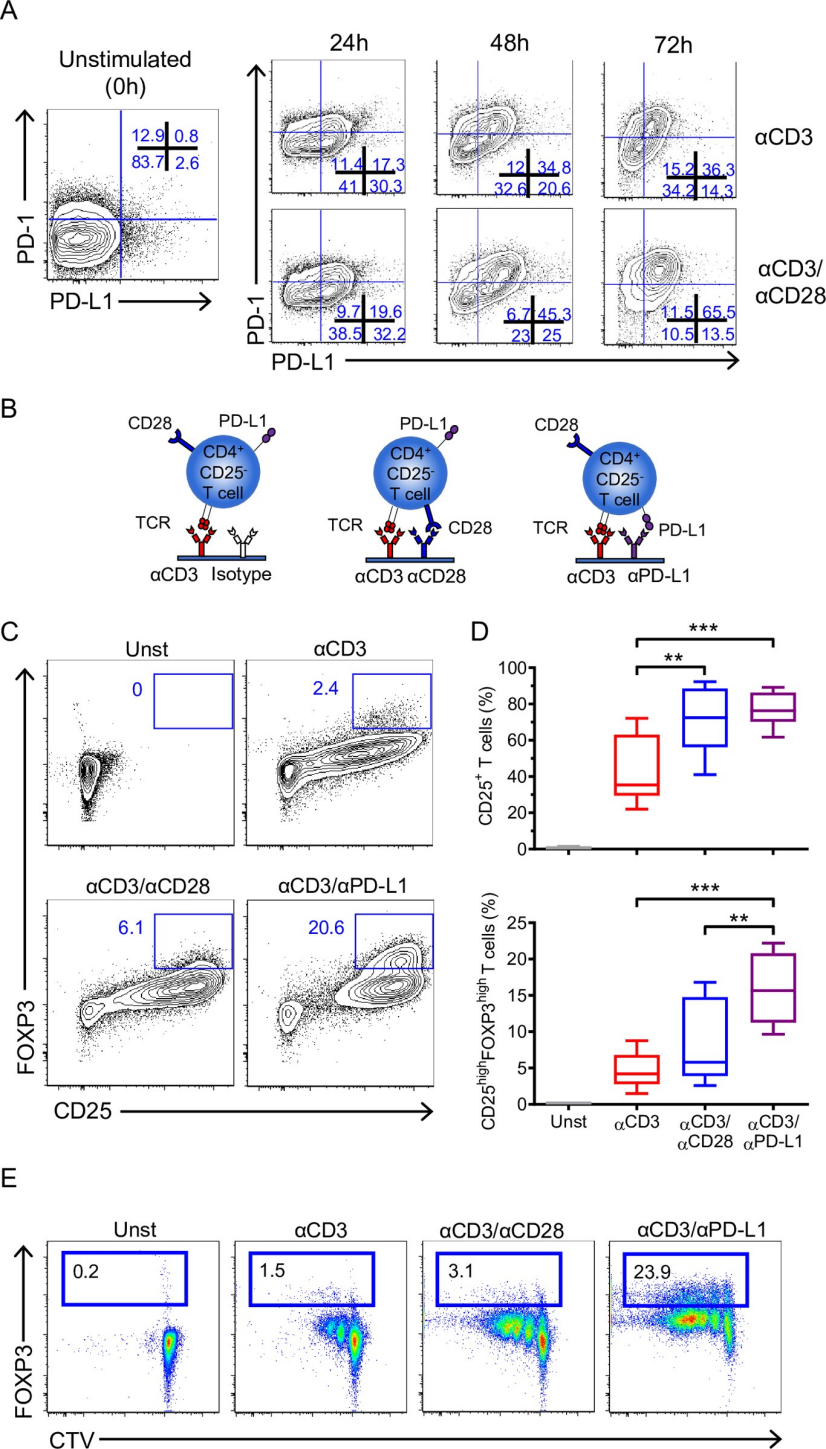
PD-L1 cross-linking on CD4^+^CD25^−^ T cells promotes their activation and proliferation. (**A**) Representative dot plots showing the expression of PD-1 and PD-L1 on freshly isolated CD4^+^CD25^−^ T cells (left panel) and upon stimulation with αCD3 or αCD3/αCD28 at different time points (24, 48, and 72 h) (right panel). (**B**) Schematic representation of different activation conditions used in our model. CD4^+^CD25^−^ T cells were stimulated as indicated in (**B**) for 72 h, and (**C**) the expression of CD25 and FOXP3 was analyzed and (**D**) quantified (*n =* 8 from 7 independent experiments). Data are represented using boxplots indicating the min and max and median; **P < 0.01 and ***P < 0.001 by RM one-way ANOVA followed by Tukey multiple comparison test. (**E**) Representative plots showing proliferation (Cell Trace Violet dye dilution) and FOXP3 expression of CD4^+^CD25^−^ T cells stimulated as in (**B)** for 72 h. Percentages of FOXP3 positive cells (box) are indicated. Data are representative of at least 2 independent experiments. Values for each data point can be found in S1 Data. Full gating strategies from representative plots are shown in S1 Gating Strategy. PD-1, Programmed cell death protein 1; PD-L1, PD-1 ligand 1; RM, repeated measures; TCR, T cell receptor.

To investigate whether PD-L1 plays a role on CD4^+^ T cells, we analyzed the expression of PD-L1 and its receptor PD-1 on freshly isolated CD4^+^CD25^−^ T cells (>96% purity) [18]. PD-1 was detected on a small subpopulation of resting CD4^+^CD25^−^ T cells, whereas PD-L1 expression was absent (**Fig 1A**). Then, their expression was analyzed after in vitro activation with plate-bound anti-CD3 antibody (αCD3) alone or in combination with anti-CD28 antibody (αCD3/αCD28). Both stimulations up-regulated PD-1 and PD-L1 after 24 h, but high expression of PD-1 and PD-L1 were observed only with αCD3/αCD28 activation after 72 h (**Fig 1A**).

We questioned whether the engagement of PD-L1 on T cells might have an effect. Thus, we activated CD4^+^CD25^−^ T cells with plate bound anti-CD3/anti-PD-L1 antibodies (αCD3/αPD-L1) and compared their phenotype to cells exposed to αCD3 or αCD3/αCD28 (**Fig 1B**). Of note, a matching isotype control antibody was coated in combination with αCD3 to keep the same concentration of each antibody on the well. After 72 h, the percentage of CD25^+^ T cells was comparable between αCD3/αPD-L1 and αCD3/αCD28 stimulations but significantly higher than αCD3 alone (means of 76.6%, 71% and 43.2%, respectively) (**Fig 1C and 1D**), suggesting an effect of PD-L1 in T cells. Cross-linking with αCD3/αPD-L1 promoted the highest percentage of CD25^high^ cells (**Fig 1C and 1D**) and up-regulated the surface molecule CTLA-4, thus inducing phenotypic characteristics common to Treg cells (**S1A and S1B Fig**). Furthermore, the total number of cells recovered was similar between the different stimulations at the end of culture (**S1C Fig**), supporting the idea that the conversion into CD25^+^FOXP3^high^ cells upon PD-L1 engagement is a functional change rather than the enrichment of a particular subset during the culture. Although most FOXP3^+^ T cells would have been in the CD25^+^ fraction that was depleted before activation, the increase in FOXP3 percentage upon αCD3/αPD-L1 stimulation could be due to the activation and expansion of a FOXP3^+^CD25^−^ T cell population. To confirm that the differentiation of CD4^+^CD25^−^FOXP3^−^ T cells into FOXP3^+^ T cells was due to de novo expression rather than to the expansion of a contaminating preexisting FOXP3^+^ T cell population in the preparation, we analyzed FOXP3 expression on cell trace violet-labeled cells. This experiment revealed that FOXP3 was up-regulated not only in dividing cells but also in nondividing cells, demonstrating that the induction of FOXP3 was indeed mediated by PD-L1 engagement and was not an artifact due to the proliferation of a contaminating FOXP3^+^ T cell population (**Fig 1E**).

Several reports have shown that suboptimal TCR stimulation favors the generation of iTregs [19]; therefore, to discard any putative artifact that could lower the αCD3 concentration due to a displacement by the αPD-L1, we combined αCD3 and αPD-L1 at different ratios and concentrations in a matrix fashion. Our results clearly showed that iTreg conversion is due to the specific cross-linking of PD-L1 and it is observed independently of the αCD3 concentration (**S1B Fig**). In addition, to rule out the possibility that the conversion was due to the specific αPD-L1 clone used, MIH1, 3 additional αPD-L1 antibodies were also tested. The results obtained with the 3 new antibodies were similar to the results with the MIH1 antibody, although some variation was seen arguable due to the different antibody affinities (**S1D Fig**).

### Signaling through PD-L1 in the absence of PD-1 engagement activates and converts CD4^+^CD25^−^ T cells into highly suppressive iTreg cells

**Fig 2:**
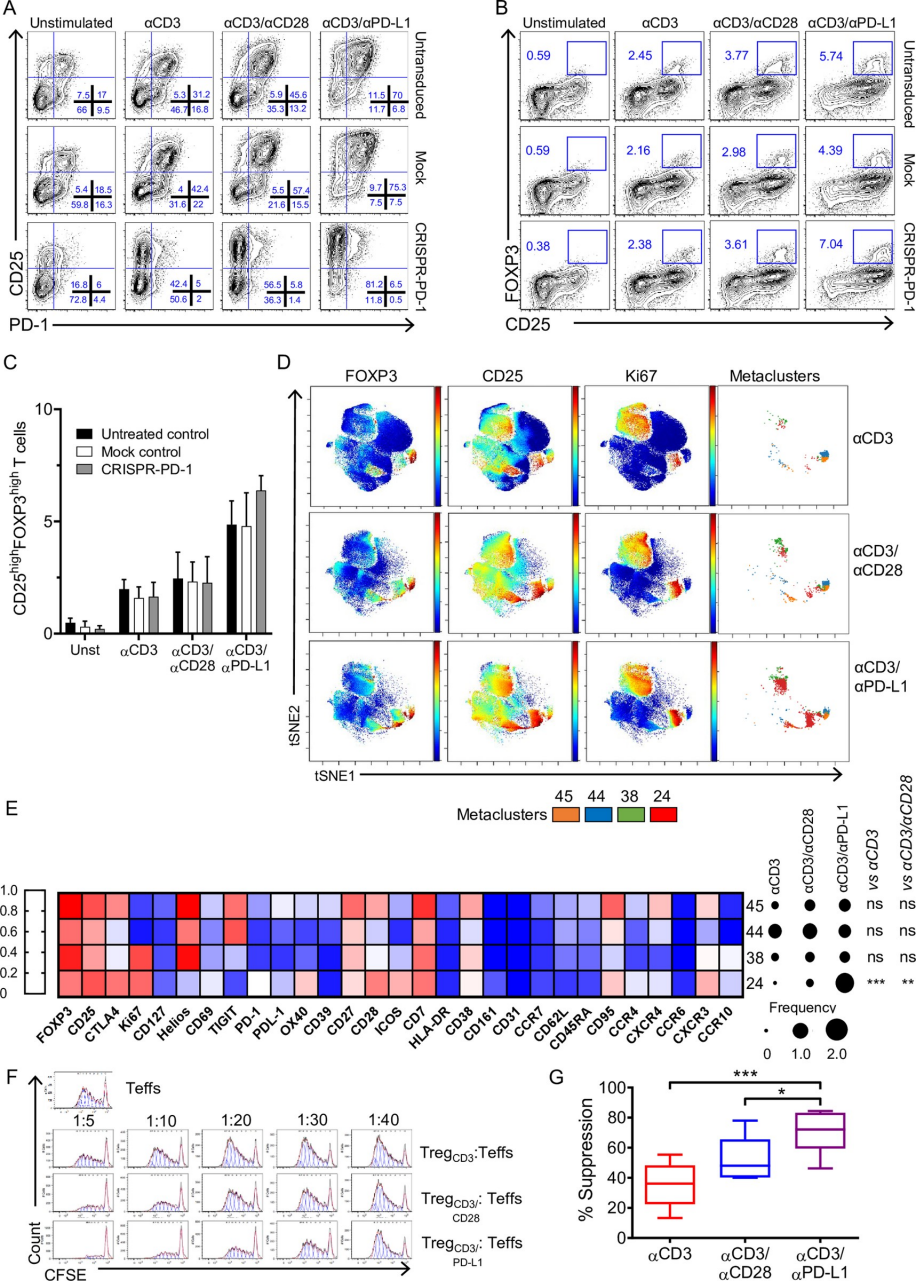
PD-L1 signaling activates and converts CD4^+^CD25^−^ into iTregs. (**A**) Representative dot plots showing CD25 and PD-1 expression on untreated control, mock-treated, and CRISPR/Cas9-PD-1-edited CD4+CD25− T cells activated for 72 h as indicated. (**B**) Representative plots showing FOXP3 and CD25 expression of untreated control, mock control, and CRISPR/Cas9-PD-1-edited CD4^+^CD25^−^ T cells activated for 72 h as indicated. **(C)** Cumulative results expressed as percentage of CD25^high^FOXP3^high^ T cells. Data are expressed as mean ± SD and are pooled from at least 2 independent experiments (*n =* 3 different donors). **(D)** Representative viSNE map of manually gated CD45^+^CD3^+^CD8^−^CD4^+^ T cells clustered using surface and intracellular markers. Shown are maps for expression of indicated markers and the CD25^high^FOXP3^high^ metaclusters from FlowSOM analysis. **(E)** Heatmap of the median expression of 30 markers across the 4 metaclusters representing the CD25^high^FOXP3^high^ cells. The color in the heatmap represents the median of 0 to 1 scaled expression values of arcsinh transformed data for each marker. Dots represent the percentages of the indicated metaclusters in CD3^+^CD8^−^CD4^+^ cells; **P < 0.01 and ***P < 0.001 by two-way ANOVA followed by Tukey multiple comparison. **(F)** Representative histograms showing CFSE dilution of effector CD4^+^ T cells (1 × 10^5^) activated with αCD3/αCD28 beads at a 40:1 (cell/bead) ratio and cultured alone or in the presence of iTreg cells at the indicated ratios (Treg/Teff) for 5 days. **(G)** Quantification of suppression at 1:20 ratio of Treg/Teff cells. (*n =* 7 from 7 independent experiments). Data are represented using boxplots indicating the min and max and median; *P < 0.05 and ***P < 0.001 by RM one-way ANOVA followed by Tukey multiple comparison test. Values for each data point can be found in S1 Data. Full gating strategies from representative plots are shown in S1 Gating Strategy. CFSE, Carboxyfluorescein succinimidyl ester; iTreg, inducible Treg; PD-1, Programmed cell death protein 1; PD-L1, PD-1 ligand 1; RM, repeated measures; Teff, effector T cell; Treg, regulatory T cell.

The results so far suggested that PD-L1 could function as a cosignaling molecule supporting TCR signaling pathways. We previously showed that FOXP3 expression could be mediated solely by CD28 stimulation independently of TCR signaling [20]. Thus, we questioned whether the engagement of PD-L1 alone could drive the expression of FOXP3 without a concurrent TCR signal. The cross-linking of PD-L1 in the absence of TCR stimulation was unable to promote T cell activation and conversion into a CD4^+^CD25^+^FOXP3^high^ T cell subpopulation (S1B Fig), indicating that TCR signal is a conditio sine qua non to induce a Treg phenotype. Previous studies have shown that the binding of PD-L1 to PD-1 expressed on T cells promotes iTreg conversion [10,11]. Thus, it is possible, although unlikely, that cross-linking PD-L1 rendered PD-1 free to interact with another ligand (e.g., PD-L2), which, in turn, leads to PD-1-mediated conversion to iTregs [10]. To exclude this possibility, we generated PD-1-deficient CD4^+^CD25^−^ T cells using CRISPR/Cas9 technology, and we stimulated them under the conditions described in **Fig 1B**. A significant reduction in the percentage of PD-1^+^ cells (average efficiency of 88%) was obtained upon CRISPR/Cas9-mediated-*PD-1* gene editing compared to mock-treated T cells (**Figs 2A and S2**) in all conditions (unstimulated, αCD3, αCD3/αCD28, and αCD3/αPD-L1). As shown in **Fig 2B and 2C** the percentage of conversion of PD-1-deficient CD4^+^CD25^−^ T cells into CD25^high^FOXP3^high^ T cells was comparable to what we observed in mock-treated T cells following αCD3/αPD-L1 stimulation, suggesting that an intrinsic signaling through PD-L1 and not through PD-1 occurred in our model.

To further define how PD-L1 signaling modulates the activation of CD4^+^CD25^−^ T cells, we used an unbiased multidimensional analysis via cytometry by time-of-flight (CyTOF). First, the overall effect of the 3 different conditions was assessed on manually gated live CD45^+^CD3^+^CD4^+^ T cells by performing a marker enrichment modeling (MEM) analysis (**S3 Fig**). As shown in **Figs 1C** and **S1**, PD-L1 engagement led to an enrichment in CD25, FOXP3, and CTLA-4. Furthermore, MEM analysis revealed that PD-1, CD69, CD28, CXCR3, CCR4, Ki67, and OX40 were also enriched upon PD-L1 stimulation compared to αCD3 and αCD3/αCD28. viSNE was used to create a map of manually gated live CD45^+^CD3^+^CD4^+^ T cells and arrange them along t-SNE (**Fig 2D**). For the identification of specific cell clusters, we used a SOM-based method, followed by a consensus clustering algorithm to cluster cells and identify specific cell subsets between the 3 activation conditions described above (**S4A and S4B Fig**). The unbiased analysis confirmed our previous observation **(Fig 1C and 1D)**, namely the engagement through PD-L1 was able to activate conventional T cells. In detail, FlowSOM identified 8 metaclusters of nonactivated cells being negative for CD25 (**S4A Fig)** showing the highest expression upon CD3 activation (**S4C Fig),** while the other 42 metaclusters included cells with intermediate and high CD25 expression (**S4A Fig**). Cells activated upon PD-L1 engagement showed a significantly higher CD25 expression (**S4D Fig**) compared to αCD3 alone, and they could be divided in 2 different groups according to the expression of Ki67. This marker has been used to distinguish between metaclusters of proliferating and nonproliferating cells. Although no significant differences were found, we observed higher percentages of CD25^+^Ki67^+^ cells **(S4E Fig)** upon PD-L1 cross-linking compared to αCD3/αCD28 and αCD3 stimulation, while the levels of CD25^+^Ki67^−^ cells **(S4F Fig)** following αCD3/αCD28 and αCD3/αPD-L1 activation were comparable compared to αCD3 alone. The analysis on FOXP3^+^ cells showed that PD-L1 ligation significantly induced more CD25^high^FOXP3^+^ cells (**S5A Fig**) compared to the other activation conditions. We identified 3 metaclusters of Ki67^+^CD25^high^FOXP3^+^ cells (**S5B Fig)** that were enriched under αCD3/αPD-L1 activation, and Ki67^−^CD25^high^FOXP3^+^ cells were significantly increased after αCD3/αPD-L1 compared to αCD3/αCD28 and αCD3 alone (**S5C Fig**). CD25^high^FOXP3^+^cells included a heterogeneous group of cells defined by 9 different metaclusters with differential expression of FOXP3 and markers involved in Treg suppression, activation, and homing (**S5D Fig**). We focused on those metaclusters expressing high levels of FOXP3 as they best represent the population reported in **Fig 1C**. The percentage of CD25^high^FOXP3^high^ cells was significantly higher after αCD3/αPD-L1 activation versus αCD3 stimulation (**S6A Fig**) and consisted of 4 metaclusters (**Fig 2E**). The CD25^high^FOXP3^high^ cells belonging to the metacluster 24 were significantly more abundant after αCD3/αPD-L1 activation compared to αCD3/αCD28 and αCD3 alone (**P < 0.01 and *P < 0.05, respectively) (**Fig 2E**). They were characterized by proliferating cells expressing high level of CTLA-4, OX40, CCR4, and ICOS markers compared to Ki67^+^CD25^high^FOXP3^high^ cells (metacluster 38) (**Figs 2E and S6B).** Furthermore, these cells had a lower expression of Helios compared to the other CD25^high^FOXP3^high^ cells subsets (**Figs 2E and S6B**). Notably, Ki67^+^CD25^high^FOXP3^high^ cells were characterized by a lower expression of TIGIT compared to the Ki67^−^CD25^high^FOXP3^high^ (metacluster 44 and 45) cells (**Figs 2E and S6B).** The differential expression of the studied markers has been confirmed using MEM analysis on metaclusters representing CD25^high^FOXP3^high^ cells (**S6C Fig)**.

Altogether, these data showed that CD3/PD-L1 signaling induces a different program in CD4^+^ T cells than CD3/CD28 stimulation resulting in the generation of different population subsets within the CD25^high^FOXP3^high^ population.

In order to investigate whether FOXP3^high^ T cells induced by PD-L1 ligation had regulatory functions, we assessed their suppressive capacity. Cells generated by αCD3/αPD-L1 cross-linking were significantly more suppressive than those generated with both αCD3 alone and αCD3/αCD28 (**Fig 2F and 2G)**. Thus, these data suggest that T cells generated upon PD-L1 cross-linking acquired a highly suppressive function and became iTregs.

### Memory T cells but not naïve T cells are converted into regulatory FOXP3^+^ CD4^+^ T cells by PD-L1 cross-linking

**Fig 3:**
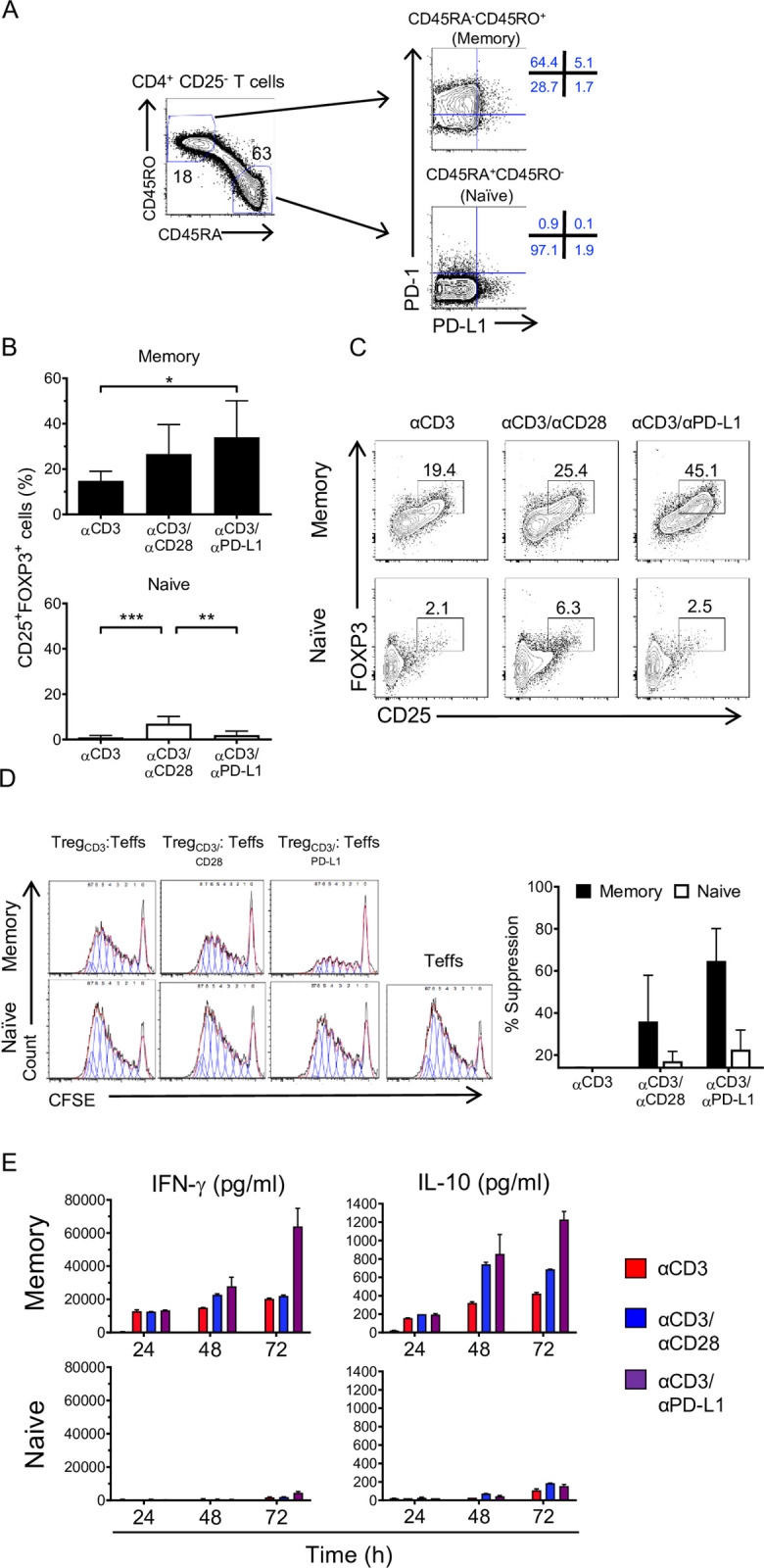
iTreg conversion is induced preferentially in memory cells upon PD-L1 engagement. (**A**) Gating strategy used to sort CD4^+^CD25^−^ T cells in naïve and memory subsets based on their CD45RO and CD45RA expression (left panel). Representative plot of sorted populations stained for PD-1 and PD-L1 expression (right panel). (**B**) Quantification of CD25 and FOXP3 expression on memory (upper panel) (*n =* 4) and naïve (bottom panel) (*n* = 7) subsets following 72 h of activation with αCD3, αCD3/αCD28, and αCD3/αPD-L1. Bars represent the mean ± SD; *P < 0.05, **P < 0.01, and ***P < 0.001 by RM one-way ANOVA followed by Tukey multiple comparison. (**C**) Representative plots showing combined analysis of FOXP3 and CD25 and expression on memory and naïve subsets under the indicated conditions. (**D**) Representative histograms showing CFSE dilution of effector CD4^+^ T cells activated with αCD3/αCD28 beads and cultured alone or in the presence of memory or naïve T cells stimulated as in (**B**) at a 1:20 (Treg/Teff) ratio for 5 days. Histograms are representative of 2 independent experiments (*n* = 3). Quantification of suppression at 1:20 ratio of Treg/Teff cells, bars represent the mean ± SD. (**E**) Absolute values of IFN-γ and IL-10 cytokine production by memory and naïve cells activated for 24, 48, and 72 h under the indicated conditions. Values for each data point can be found in S1 Data. Full gating strategies from representative plots are shown in S1 Gating Strategy. CFSE, Carboxyfluorescein succinimidyl ester; IFN-γ, interferon gamma; IL-10, interleukin 10; iTreg, inducible Treg; PD-1, Programmed cell death protein 1; PD-L1, PD-1 ligand 1; RM, repeated measures; Teff, effector T cell; Treg, regulatory T cell.

Previous in vitro and in vivo studies have shown that generation of iTreg cells involves the peripheral activation of naïve T CD4^+^ cells in the presence of TGF-β and IL-2 [21]. However, the capacity of memory T cells to be converted into FOXP3^+^ iTreg is still controversial [22,23]. Thus, we evaluated the contribution of the CD4^+^CD25^−^ T cell subpopulations, i.e. CD4^+^CD25^−^CD45RA^−^CD45RO^+^ (memory) T cells and CD4^+^CD25^−^CD45RA^+^CD45RO^−^ (naïve) T cells, to the generation of iTreg cells upon PD-L1 engagement. Naïve and memory T cells were sorted according to CD45RA and CD45RO expression, and PD-1 and PD-L1 expression were analyzed by flow cytometry before (**Fig 3A**) and after stimulation with αCD3 and αCD3/αCD28 (**S7 Fig**). After stimulation with αCD3 and αCD3/αCD28, the expression of PD-L1 and PD-1 on naïve T cells remained low and rapidly became undetectable (**S7 Fig**). In contrast, on resting memory T cells (**Fig 3A**), PD-1 was highly expressed, while low levels of PD-L1 were detected. Following stimulation, an up-regulation of both PD-L1 and PD-1 on memory T cells occurred over time (**S7 Fig**). This result suggested that memory T cells had become more prone to PD-L1 stimulation. In fact, cross-linking by αCD3/αPD-L1 was able to induce conversion of memory T cells to CD25^+^FOXP3^+^ Tregs (**Fig 3B, upper panel**), while naïve T cells stimulated under the same conditions failed to convert into CD25^+^FOXP3^+^ T cells (**Fig 3B, bottom panel**). In contrast to αCD3/aPD-L1, αCD3/αCD28 stimulation induced CD25 and FOXP3 expression in both naïve and memory T cells (**Fig 3B and 3C**), suggesting that the negligible expression of PD-L1 on naïve T cells and the failure to up-regulate it upon stimulation are, at least in part, the cause for the lack of response to αCD3/αPD-L1 cross-linking. Upon PD-L1 engagement, memory T cells showed high suppressive capacity (**Fig 3D**), supporting their phenotypical and functional identity as iTregs.

**Fig 4:**
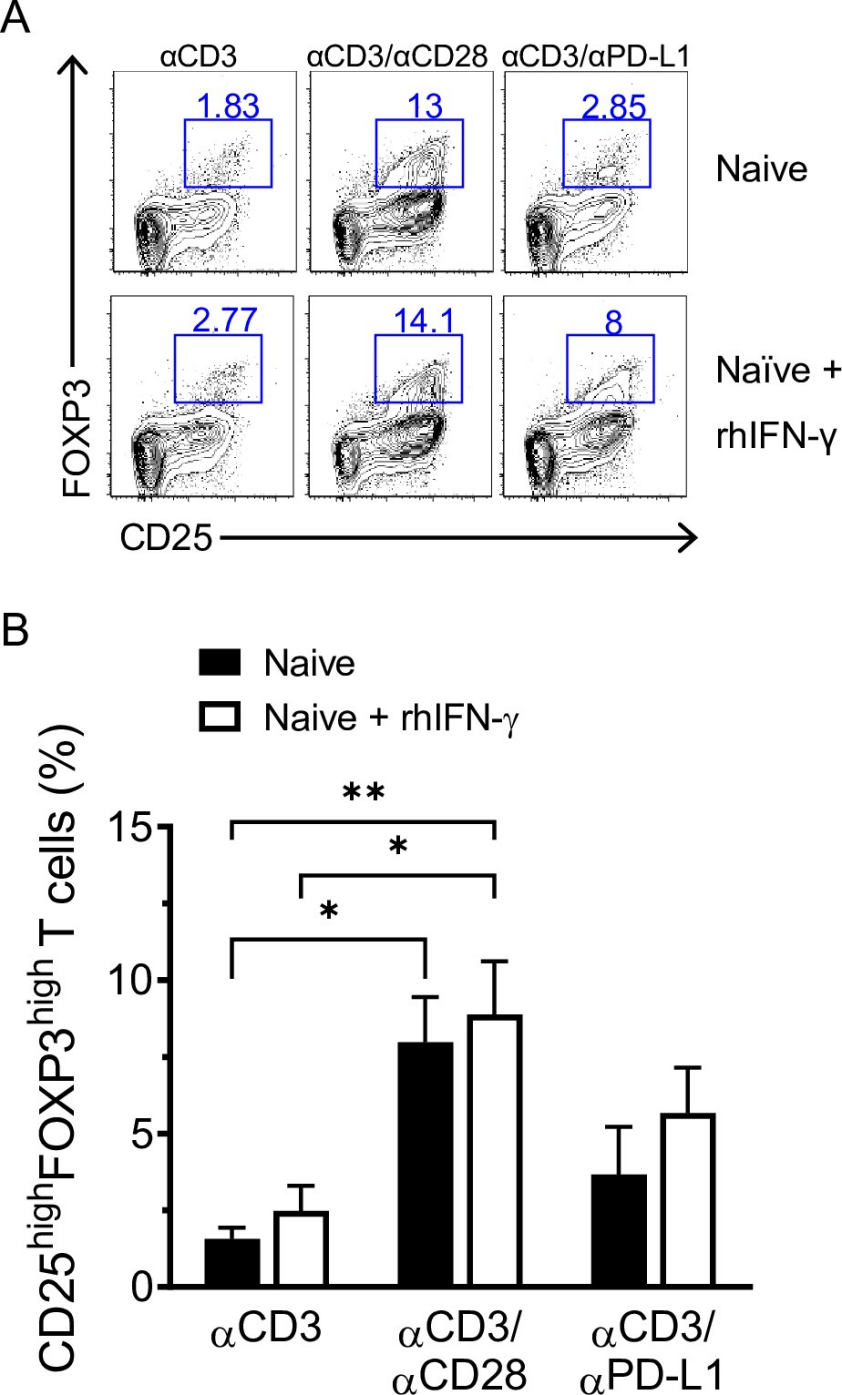
PD-L1 costimulation promotes iTreg conversion of naïve T cells in the presence of IFN-γ. (**A**) Representative dot plots showing CD25 and FOXP3 expression after 72 h of culture of naïve CD4^+^CD25^−^ T cells isolated from healthy donors in the presence or absence of rhIFN-γ. (**B**) Cumulative results expressed as percentage of CD25^high^FOXP3^high^ T cells. Data are expressed as mean ± SD and are pooled from at least 2 independent experiments (*n =* 5). *P < 0.05 and **P < 0.01 by two-way ANOVA followed by Tukey multiple comparison. Values for each data point can be found in S1 Data. Full gating strategies from representative plots are shown in S1 Gating Strategy. IFN-γ, interferon gamma; iTreg, inducible Treg; PD-L1, PD-1 ligand 1; rhIFN-γ, recombinant human interferon gamma.

Since IFN-γ is able to induce the up-regulation of PD-L1 [24] and IL-10 is a well-known mediator of Treg suppression [25], we analyzed the cytokines produced by memory and naïve T cells following stimulation. We observed an increase of IL-10 production that has been reported to correlate with a suppressive role during the resolution phase following inflammation [26]. Memory T cells produced high levels of IFN-γ, particularly after cross-linking with αCD3/αPD-L1 (**Fig 3E**), while naïve T cells produced insignificant levels of IFN-γ regardless of the stimulus (**Fig 3E**). This suggests a positive feedback loop that sustains the expression of PD-L1 on those memory FOXP3^+^ iTregs that keep the balance between tolerogenic and inflammation to limit immune-mediated side effects. Overall, these data indicate that, in our system, PD-L1 engagement preferentially converts memory T cells into functional iTregs. Moreover, since naïve T cells do not convert under αCD3/αPD-L1 condition, but the unfractionated CD4^+^CD25^−^ T cells behave as memory T cells, we asked whether IFN-γ produced by memory T cells contributed to the conversion of naïve T cells by up-regulating PD-L1. Like memory T cells, naïve T cells stimulated with αCD3/αPD-L1 in the presence of recombinant human IFN-γ showed an increase of CD25^high^FOXP3^high^ frequency although not statistically significant compared to naïve T cells without IFN-γ, while naïve T cells stimulated with αCD3 alone or αCD3/αCD28 in the presence of IFN-γ remained unchanged (**Fig 4A and 4B**).

### PD-L1 ligation on memory T cells drives iTreg conversion by concomitantly down-regulating AKT/mTOR/S6 and TCR/MAPK signaling cascades

**Fig 5:**
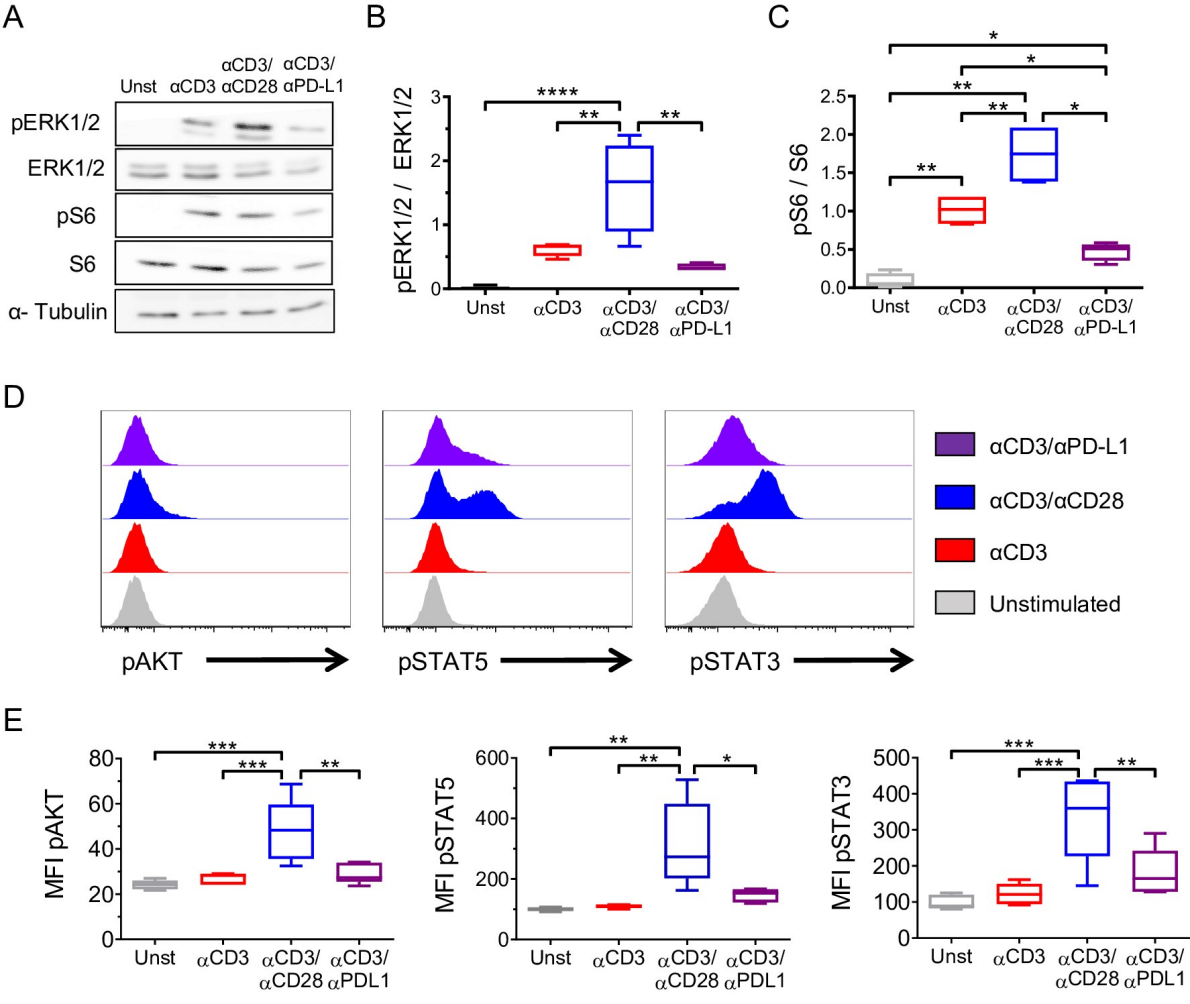
Cosignaling through PD-L1 promotes iTreg conversion by modulating AKT/mTOR/S6 and TCR/MAPK pathways. (**A**) Representative immunoblot of pERK1/2, ERK1/2, pS6, and S6 in memory T cells upon 15 min of activation under the indicated conditions. α-Tubulin was used as loading control. Western blot quantification of (**B**) ERK1/2 (pERK1/2/ERK ratio) and (**C**) S6 (pS6/S6 ratio) activation status are shown (*n =* 6, 6 independent experiments). Data are represented using boxplots indicating the min and max and median; *P < 0.05, **P < 0.01, *** *P* < 0.001, and ****P < 0.0001 by RM one-way ANOVA followed by Tukey multiple comparison. (**D**) Representative flow cytometry histograms showing the MFI of pAKT, pSTAT3, and pSTAT5 following 48 h of stimulation under the indicated conditions. (**E**) Quantification of pAKT, pSTAT5, and pSTAT3 MFI (*n* = 6). Data are represented using boxplots indicating the min and max and median; *P < 0.05, **P < 0.01, and ***P < 0.001 by RM one-way ANOVA followed by Tukey multiple comparison test. Values for each data point can be found in S1 Data. Full gating strategies from representative plots are shown in S1 Gating Strategy. Full blots are available in S1 Raw Images. ERK1/2, total ERK; iTreg, inducible Treg; MAPK, mitogen-activated protein kinase; MFI, mean fluorescence intensity; pAKT, phospho-AKT; PD-L1, PD-1 ligand 1; pERK1/2, phospho-ERK1/2; pS6, phopho-S6; pSTAT3, phospho-STAT3; pSTAT5, phospho-STAT5; RM, repeated measures; S6, total S6; TCR, T cell receptor.

Cytokines, TCR signaling, costimulatory/coinhibitory molecules, and metabolic changes are known to influence the generation of Tregs in the periphery. Since several reports have shown that suboptimal TCR stimulation favors the generation of iTregs [19], we investigated whether PD-L1 ligation resulted in a modulation of the TCR signaling cascade. To assess this, we isolated CD4^+^CD25^−^ memory T cells and stimulated them with αCD3 alone for 24 h to allow the up-regulation of PD-L1, then cells were activated under the 3 conditions for 15 min. Although both αCD3/αPD-L1 and αCD3/αCD28 conditions induced similar levels of CD25 expression (**Fig 1D),** western blot analysis revealed that αCD3/αCD28 simulation increased significantly ERK1/2 phosphorylation (**Fig 5A and 5B**), whereas αCD3/αPD-L1 engagement had the opposite effect showing a slightly attenuated TCR/MAPK signaling pathway compared to αCD3 alone, as shown by the lower phosphorylation of ERK1/2 (**Fig 5A and 5B**). In addition, it is well established that the mTOR pathway is required in conventional T cells but dispensable by Tregs [27,28]. mTOR has as a downstream target p70 S6 kinase which, in turn, phosphorylates the S6 ribosomal protein. Thus, we evaluated the AKT/mTOR/S6 pathway using phosphorylated S6 (pS6) as an indicator of the mTOR pathway activation status. As shown in **Fig 5A** and **5C**, high levels of pS6 were obtained with αCD3/αCD28 cross-linking, while pS6/S6 ratio after αCD3/αPD-L1 cross-linking was not only significantly lower than after αCD3/αCD28 stimulation but also 50% lower than following αCD3 stimulation alone (**Fig 5A and 5C**). Furthermore, αCD3/αCD28 stimulation led to the highest level of pAKT, while PD-L1 ligation showed similar levels of pAKT compared to both αCD3 alone and unstimulated cells (**Fig 5D and 5E, left panel**). Altogether, these results confirmed that PD-L1 delivers a signal in T cells. Moreover, PD-L1 signal is different than the signal induced by both αCD3 and αCD3/αCD28 stimulations, and it is indicative of a modulation of the central metabolic AKT/mTOR pathway associated with the acquisition of a regulatory phenotype [29].

Since other signals play a role in the conversion and function of Tregs, we sought to analyze STAT5 and STAT3 phosphorylation status that are known to participate in Treg development and function [30,31]. STAT3 and STAT5 phosphorylation were significantly higher when T cells were stimulated with αCD3/αCD28 compared to αCD3 stimulation alone or in combination with PD-L1 **(Fig 5D and 5E**). While αCD3/αPD-L1 cross-linking showed a consistent and reproducible increase in STAT3 and STAT5 phosphorylation, it did not reach statistical significance compared to αCD3 stimulation alone. (**Fig 5D and 5E**). Collectively, these data suggest an intrinsic signaling through PD-L1 that favors the conversion of memory T cells into a regulatory phenotype by reducing the strength of the TCR signaling, antagonizing the AKT/mTOR pathway, and increasing STAT3 and STAT5 phosphorylation to intermediate levels.

### PD-L1 engagement fails to induce a regulatory phenotype in rheumatoid arthritis patients

Previous studies have reported that T cells in rheumatoid arthritis (RA) patients have ERK phosphorylated at higher levels than healthy donors and that these levels are further increased upon activation [32,33]. It has been suggested that the dysregulation in ERK activity in RA patients predisposes to the activation of autoreactive T cells favoring disease progression [32]. Since PD-L1 ligation led to a combined reduction of ERK and S6 phosphorylation in healthy donors, we sought to investigate whether PD-L1 engagement was able to modulate T cell signaling in RA patients.

**Fig 6:**
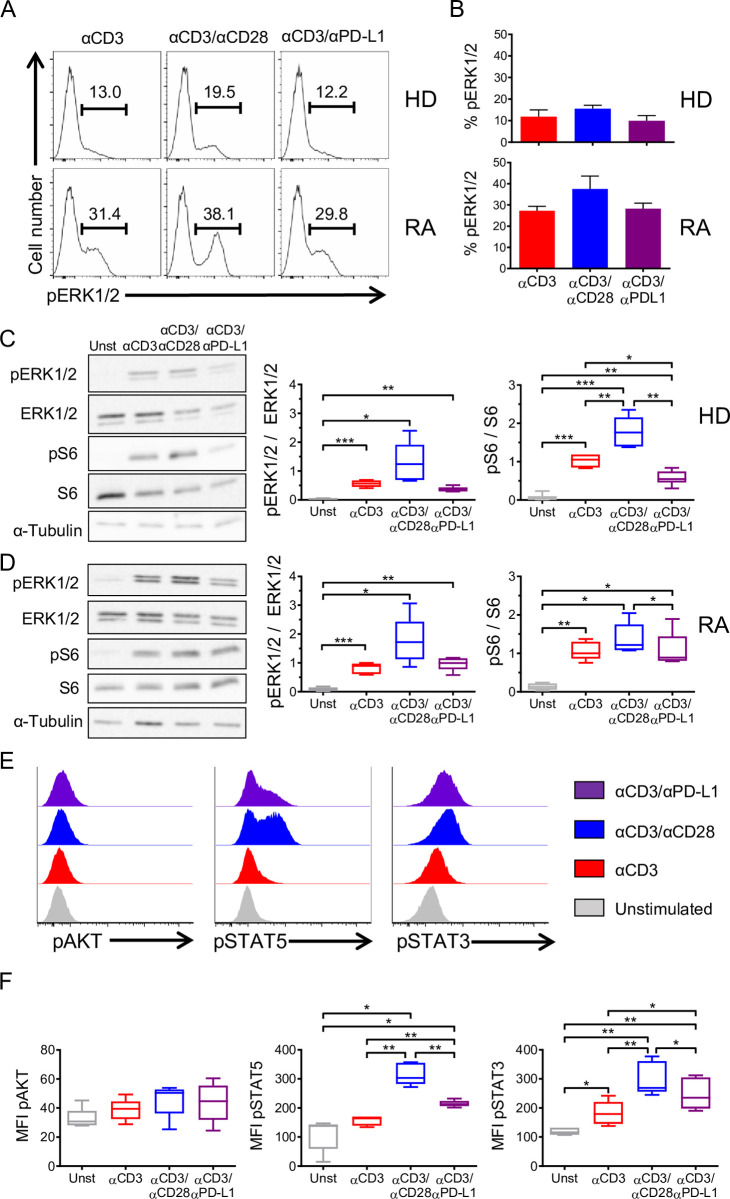
PD-L1 engagement fails to convert CD4^+^CD25^−^ T cells from RA patients into iTreg cells. Representative flow cytometry histograms (**A**) and bar charts (**B**) showing the percentage of pERK1/2 in HDs and RA patients upon the indicated stimulation. (**C**) Representative immunoblot of pERK1/2, ERK1/2, pS6, and S6 in CD4^+^CD25^−^ T cells isolated from HDs upon 15 min of activation under the indicated conditions. α-Tubulin was used as loading control (*n =* 6). Data are represented using boxplots indicating the min and max and median; *P < 0.05, **P < 0.01, and ***P < 0.001 by RM one-way ANOVA followed by Tukey multiple comparison. (**D**) Representative immunoblot of pERK1/2, ERK1/2, pS6, and S6 in CD4^+^CD25^−^ T cells isolated from RA patients (*n =* 6). Data are represented using boxplots indicating the min and max and median; *P < 0.05, **P < 0.01, and ***P < 0.001 by RM one-way ANOVA followed by Tukey multiple comparison test. (**E**) Representative flow cytometry histograms showing the MFI of pAKT, pSTAT3, and pSTAT5 of RA CD4^+^CD25^−^ T cells following 48 h of stimulation under the indicated conditions. (**F**) Quantification of pAKT, pSTAT5, and pSTAT3 MFI shown in (E) (*n =* 5). Data are represented using boxplots indicating the min and max and median; *P < 0.05 and **P < 0.01 by RM one-way ANOVA followed by Tukey multiple comparison test. Values for each data point can be found in S1 Data. Full gating strategies from representative plots are shown in S1 Gating Strategy. Full blots are available in S1 Raw Images. ERK1/2, total ERK; HD, healthy donor; MFI, mean fluorescence intensity; pAKT, phospho-AKT; pERK1/2, phospho-ERK1/2; pS6, phopho-S6; pSTAT3, phospho-STAT3; pSTAT5, phospho-STAT5; RA, rheumatoid arthritis; RM, repeated measures; S6, total S6.

**Fig 7:**
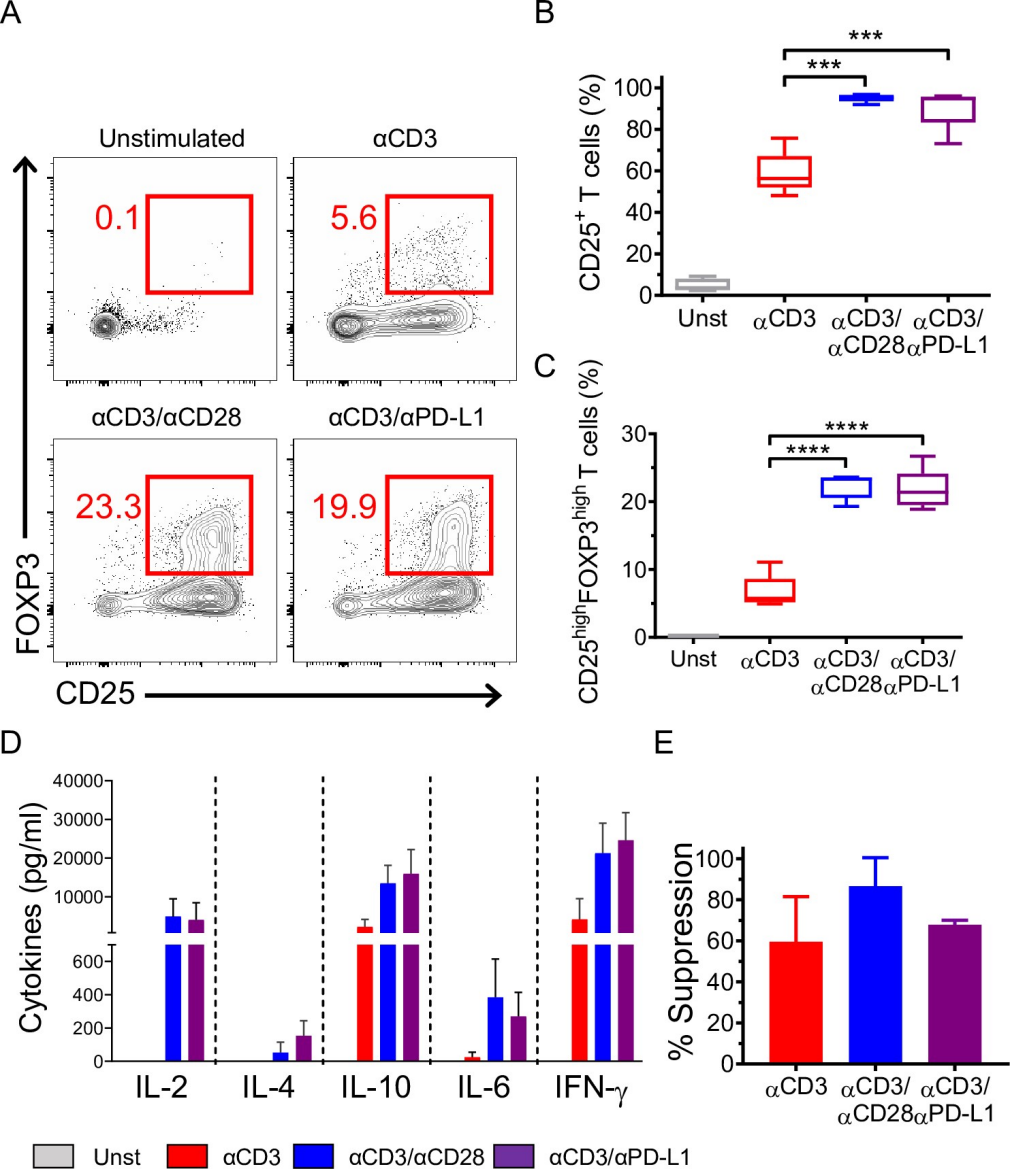
Phenotype, cytokine production, and suppressive ability of RA CD4^+^CD25^−^ T cells upon PD-L1 engagement. (**A**) Representative dot plots showing CD25 and FOXP3 expression after 72 h of culture of CD4^+^CD25^−^ T cells isolated from RA patients under the conditions indicated (left panel). (**B**, **C**) Quantification of CD25 and FOXP3 expression. Data are represented using boxplots indicating the min and max and median and are pooled from at least 2 independent experiments (*n* = 5); ***P < 0.001 and ****P < 0.0001 by RM one-way ANOVA followed by Tukey multiple comparison. (**D**) Absolute values of cytokine production by RA CD4^+^CD25^−^ T cells activated under the indicated conditions. (**E**) Suppression of RA CD4^+^CD25^−^ T cells at 1:20 ratio. Data are expressed as mean ± SD and are pooled from at least 2 independent experiments (*n* = 3). Values for each data point can be found in S1 Data. Full gating strategies from representative plots are shown in S1 Gating Strategy. PD-L1, PD-1 ligand 1; RA, rheumatoid arthritis; RM, repeated measures.

CD4^+^CD25^−^ T cells from RA patients were stimulated with αCD3, αCD3/αCD28, and αCD3/αPD-L1 following the same protocol used for healthy donors. As expected, the level of pERK in RA patients was more than 2-fold higher than in healthy individuals in all stimulatory conditions (**Fig 6A and 6B**). Healthy donor CD4^+^CD25^−^ T cells cross-linked with αCD3/αPD-L1 showed a significant reduction in S6 phosphorylation compared to αCD3 and αCD3/αCD28 (**Fig 6C**), whereas in RA patients, cross-linking with αCD3/αPD-L1 resulted in similar levels of S6 phosphorylation to those seen in response to αCD3 stimulation alone (**Fig 6D**). In addition, T cells isolated from RA patients showed comparable levels of AKT phosphorylation, regardless of the stimulus used (**Fig 6E and 6F**), and it was higher than the observed levels in healthy donors (**Fig 5D and 5E**). Altogether, this indicates that PD-L1 engagement on CD4^+^CD25^−^ T cells from RA patients is unable to down-modulate both the TCR/MAPK signaling and AKT/mTOR pathway. Furthermore, both STAT5 and STAT3 phosphorylation reached the highest levels when stimulating with αCD3/αCD28 on CD4^+^CD25^−^ T cells from RA patients (**Fig 6E and 6F**) as in T cells from healthy donors (**Fig 5D and 5E**). In contrast to healthy donors, RA-derived T cells showed significantly higher levels of STAT5 and STAT3 phosphorylation when cross-linked with αCD3/αPD-L1 compared to αCD3 alone (**Fig 6E and 6F**). Overall, these findings suggest that T cells from RA patients may be incapable of acquiring a regulatory phenotype. Therefore, we analyzed the phenotype of CD4^+^CD25^−^ T cells after αCD3/αPD-L1 engagement. Due to the high basal level of pERK, cells had a lower threshold of activation that was evidenced by the strong up-regulation of CD25 under all experimental conditions, particularly when cross-linked with αCD3/αCD28 and αCD3/αPD-L1 with almost 100% of cells becoming CD25^+^ (**Fig 7A and 7B**). We also found a similar percentage of CD25^high^FOXP3^high^ cells following αCD3/αPD-L1 and αCD3/αCD28 cross-linking (**Fig 7A and 7C**) that was in line with the similar pattern of cytokine production (**Fig 7D**). Since αCD3/αCD28 and αCD3/αPD-L1 induced a similar phenotype ex vivo, we next assessed their suppressive capacity. CD25^+^FOXP3^high^ cells generated from RA patients generated with either αCD3/αCD28 or αCD3/αPD-L1 showed a suppressive behavior similar to activated T cells (**Fig 7E**) [34]. Furthermore, CD4^+^CD25^+^ T cells from RA patients proliferated extensively in a suppression assay compared to healthy donor cells, suggesting that the inhibition observed may be due to the overgrowth of this population of cells during the coculture (**S8 Fig**) as already reported by others [35]. Altogether, our results suggest a defect on CD4^+^ T cells from RA patients where PD-L1 engagement was unable to induce a phenotype different than that of CD28 costimulation that could be a contributing cause to diseases progression.

## Discussion

PD-L1 is up-regulated on T cells after activation, but its role on T cells remains elusive. Here, we describe how the engagement of PD-L1 on effector T cells functions as a cosignal to promote a regulatory phenotype. Our study demonstrates that PD-L1, like the canonical costimulatory molecule CD28, supports TCR signaling for an optimal induction of T cell activation and proliferation. However, unlike CD28, cosignaling through PD-L1 induced a unique CD25^high^FOXP3^high^ population that is highly suppressive.

The effects of PD-1 signaling on T cells have been extensively studied, particularly in the context of the cancer microenvironment where the engagement of PD-L1 expressed by tumor cells inhibits T cell response against the tumor. PD-L1 is used in cancer as a biomarker of poor prognosis and immune-checkpoint inhibitor therapies with anti-PD-1 blocking antibodies are currently trialed with encouraging results [36]. However, blocking PD-1/PD-L1 axis is not without pitfalls and adverse effects such as autoimmune diseases are among the most frequently described [37]. The rationale of using an anti-PD-1 blocking antibody is to avoid a negative signal into the T cells when engaging PD-L1 on tumor cells [38,39]. Anti-PD-L1 blocking antibodies aiming at disrupting this interaction could have an additional effect since PD-L1 was described to deliver a survival signal to tumor cells [15] that confers resistance to IFN-mediated toxicity [16].

The data from this study may help to explain a long-standing conundrum that antigen-presented by human activated CD4^+^ T cells led to unresponsiveness and even a suppressive phenotype [40]. In humans, activated T cells express the major histocompatibility complex class II [41,42] and other costimulatory (e.g., CD80) and coinhibitory (e.g., CTLA-4) molecules [43–45]. We have shown that the recognition of HLA-DR–peptide complexes on activated T cells by other activated T cells led to anergy, and this occurs despite the expression of costimulatory molecules (CD80 and CD86) on the same T cells [46]. Our results suggest that the expression of PD-1 and PD-L1 on activated T cells could contribute to the induction of anergy during T–T interaction. However, whether the interaction of PD-1:PD-L1 during T:T presentation plays an important role in vivo in inhibiting an immune response by inducing CD4^+^ Tregs could not be tested in a murine model due to the lack of MHC-II expression on activated mouse T cells [47].

High-dimensional data analysis suggests that PD-L1 engagement generated a specific subset of cells (metacluster 24) with low expression of Helios and high levels of ICOS, CD28, CCR4, and CTLA-4 compared to the other CD25^high^FOXP3^high^ cells. T cells with a similar phenotype (CD28^high^ ICOS^high^ and CTLA-4^high^ Helios^−^) have been described as memory Tregs [48]. These cells encompass peripheral iTregs with methylated FOXP3 locus, which produce IL-10, and are highly suppressive ex vivo and display plasticity under inflammatory conditions. Our results indicate that PD-L1 engagement induces a defined subset of iTregs and the enrichment on the metaclusters mentioned above could help to define the markers to further investigate the role of this population in healthy donors resolving an inflammation and understand their role in chronic infections, transplantation, and within tumors.

**Fig 8:**
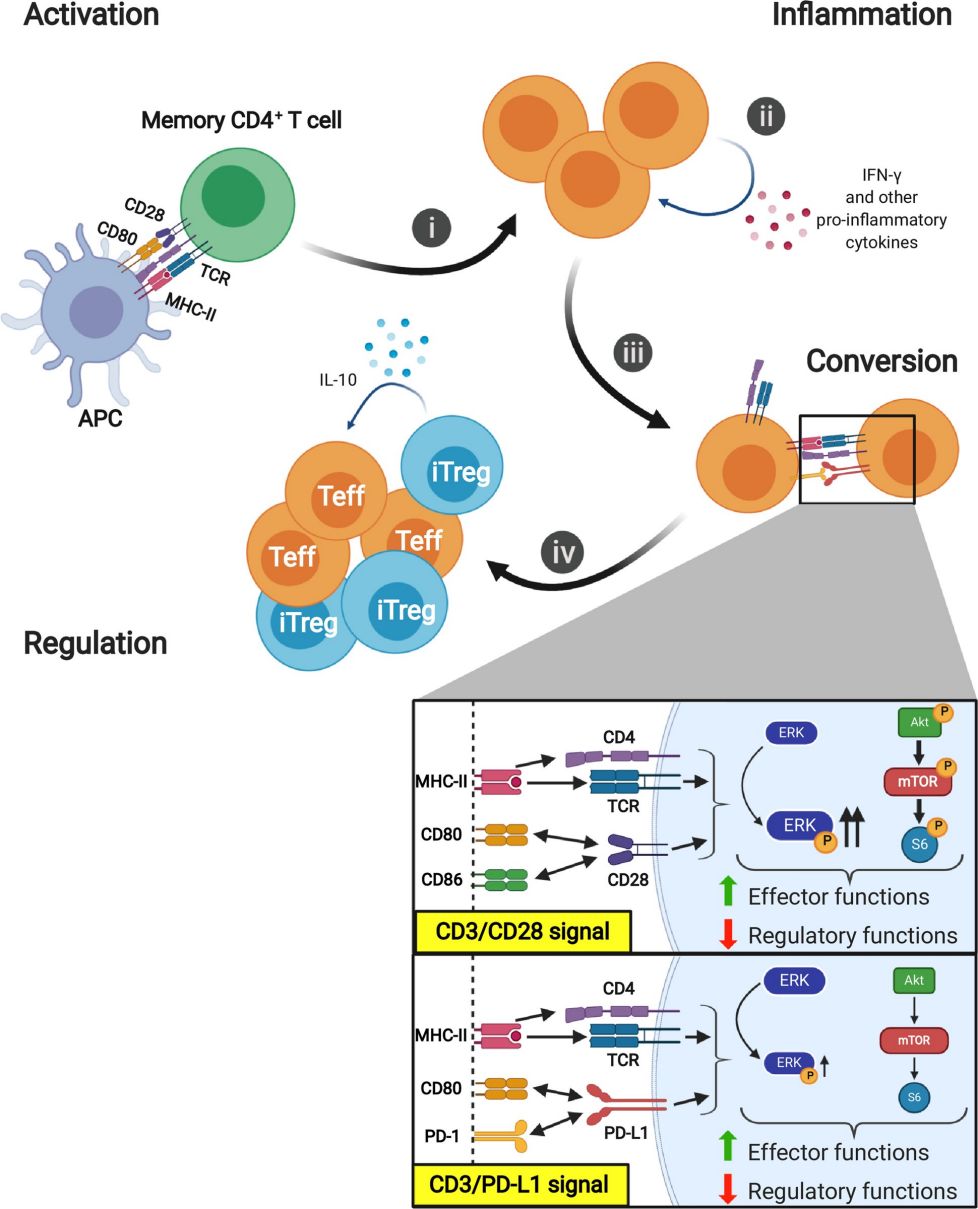
Proposed role of PD-L1 on contraction of activated effector T cells. **(i) Activation:** Following TCR/CD28 stimulation of memory T cells, MAPK/ERK pathway and AKT/mTOR/S6 pathway are activated. **(ii) Inflammation:** Activated T cells migrate to the sites of inflammation where they produce large amount of IFN-γ that induce and sustain PD-L1 expression. **(iii) Conversion:** PD-L1 ligation on effector T cells induces a reverse signal that reduces ERK phosphorylation and attenuates the AKT/mTOR/S6 pathway leading to the conversion into iTregs by up-regulating FOXP3 and CTLA-4 expression and secretion of IL-10. **(iv) Regulation:** iTregs exert their regulatory functions on effector cells controlling the inflammation. These transient iTreg cells restrain the magnitude of the immune response bringing back the immune system to homeostasis. Schematic representations were created with Biorender.com. CTLA-4, cytotoxic T-lymphocyte–associated antigen 4; IFN-γ, interferon gamma; IL-10, interleukin 10; iTreg, inducible Treg; MAPK, mitogen-activated protein kinase; PD-L1, PD-1 ligand 1; TCR, T cell receptor.

The fact that only memory T cells were susceptible to conversion into iTregs upon PD-L1 engagement suggests a potential role of PD-L1 in fine-tuning T cell effector activity in peripheral tissues and subsequently in limiting and resolving the immune response in an inflammatory environment (**Fig 8**). In fact, it is plausible that the preferential engagement of PD-L1 and the resulting conversion of memory effector into Tregs is an additional level of control required to restore the equilibrium of the immune system after the insult occurs preventing autoimmunity.

Notably, several groups have shown that PD-1/PD-L1 axis contributes to peripheral tolerance by promoting iTreg conversion [10,49]. However, all these studies have exclusively focused on the signaling pathway downstream PD-1 receptor in T cells. Other studies have shown that a reverse signaling could occur for another PD-L1 partner, i.e., CD80 [50–52]. Our results demonstrate that PD-L1 also can signal on T cells inducing a regulatory phenotype by reducing TCR-mediated signals. In line with this, Gagliani and colleagues have recently shown that the *trans*-differentiation of Th17 cells into Tregs was induced when the signals through the TCR were inhibited showing that this event led to a resolution of the ongoing immune response [53]. Furthermore, another study in humans has shown that FOXP3^+^ Tregs were generated continuously from the CD4^+^CD45RO^+^CD25^−^FOXP3^−^ T cell pool. However, these Tregs disappeared rapidly as they were highly susceptible to apoptosis upon antigen removal and immune response clearance [54]. Similar results obtained in vivo with murine T cells have shown that in contrast to high level, low levels of TCR signals promoted iTreg generation in the periphery [55]. Other experiments performed in vitro demonstrated that prolonged TCR signaling on murine T cells antagonized the induction of FOXP3 through the AKT/mTOR signaling cascade [10,56].

Our results show that PD-L1 engagement attenuated the activation of AKT/mTOR/S6 and TCR/MAPK pathways during the conversion of effector T cells into iTreg cells. In addition, the combinatorial inhibition of those pathways, which converge on S6 activation, has been shown to be effective in inhibiting memory T cell response [57,58]. Furthermore, we showed that PD-L1 signaling led to a slight increase of STAT3 and STAT5 phosphorylation. The role of STAT5 signaling in the generation of iTreg cells is well documented [30]. Although high levels of STAT3 phosphorylation are associated with an inflammatory phenotype, low levels of pSTAT3 are necessary for Treg generation [59]. We have previously described a Treg population which expresses STAT3 [60], and more recently, it has been shown that FOXP3 acts as a cotranscription factor with STAT3 leading to IL-10 production [61], consistent with the high levels of IL-10 produced by αCD3/αPD-L1 generated cells. Recently, Diskin and colleagues have shown that PD-L1 engagement up-regulated STAT3 signaling in CD4^+^ T cells that mitigates a T helper 1 and 2 polarization while partially promoting a T helper 17 differentiation [17]. However, it is important to highlight that their experimental setup differs from ours in many aspects, i.e., we used purified CD4^+^CD25^−^ T cells as starting point, while Diskin and colleagues used total or CD4^+^ T cells. They cultured T cells in plates coated with aCD3/aCD28 Abs with a monomeric soluble PD-1-Fc that is capable of blocking PD-L1.

In our study, we have observed that PD-L1 engagement on T cells from RA patients was neither able to modulate ERK signal, nor the mTOR pathway. Nevertheless, a strong phosphorylation of both STAT3 and STAT5 was induced, suggesting that the ligation of PD-L1 in RA patient T cells is not able to limit inflammation and may be in part responsible for the development of autoimmune responses. Based on this observation, it is possible that the abrogation of PD-L1 signaling pathway might contribute to the onset of rheumatic immune-related side effects observed in neoplastic patients receiving PD-1/PD-L1 blocking therapy [37,62]. However, the number of RA patients in our study is limited, and a larger cohort will be needed to confirm our observation.

In conclusion, our data suggest that PD-L1 engagement may play a crucial role in regulating the immune response and for this reason merits further exploration and may reveal therapeutic opportunities.

## Materials and methods

### Blood samples

Peripheral blood was obtained from anonymized leukocyte cones supplied by the National Blood Transfusion Service (NHS Blood and Transplantation, Tooting, London, UK). After obtaining written informed consent, blood samples and clinical/demographic data were collected from rheumatoid arthritis patients (**S1 Table**) recruited into the Pathobiology of Early Arthritis Cohort (PEAC) or into the Experimental Medicine and Rheumatology Biobank. Both studies received the approval from the local ethic committees (Rec. No. 05/Q0703/198 and 07/Q0605/29, respectively).

### T cells isolation

RosetteSep Human CD4^+^ T cell enrichment cocktail (STEMCELL Technologies, Canada) was used to purify CD4^+^ T cell fraction, and CD25 Microbeads II (Miltenyi Biotec, Germany) were used to separate CD4^+^CD25^−^ T cells from CD4^+^CD25^+^ T cell fraction according to manufacturer’s instructions. The purity of CD4^+^CD25^−^ cells was between 96% to 98%. To isolate CD4^+^CD25^−^CD45RO^−^ naïve T cells and CD4^+^CD25^−^CD45RO^+^ memory T cells, T CD4^+^ cells were stained with anti-CD4, anti-CD25, anti-CD45RA, and anti-CD45RO and sort on a 3 lasers FACS-ARIA high-speed cell sorter (BD Biosciences, USA).

### T cell activation and cytokine analysis

CD4^+^CD25^−^ T cells were stimulated with 5 μg/mL plate-bound anti-CD3 Ab (clone OKT3, eBioscience, USA) in combination with 10 μg/mL plate-bound anti-CD28 Ab (clone CD28.2, BioLegend, USA) or 10 μg/mL plate-bound anti-PD-L1 Ab (CD274, clone MIH1, eBioscience or when specified Biolegend clone 29E.2A3 and R&D research grade biosimilar Atezolizumab and Durvalumab clones Hu124 and Hu125, respectively) or IgG1 isotype control Ab (clone P3.6.2.8.1, eBioscience). Cells were activated for 72 h in the absence of exogenous IL-2, in a 48-well plate (VWR) at the density of 10^6^/mL in X-VIVO15 medium (Lonza, Switzerland) supplemented with heat inactivated 5% human AB serum (Biosera, France). Supernatants were used to detect human T cells cytokine production using LEGENDplex Human Th-Cytokine Assay (BioLegend, USA) following manufacturer’s instructions. Cytokines were acquired on a FACSCanto II (BD Bioscience). Data analysis was carried out on LEGENDplex Data Analysis Software.

### Flow cytometry

Following activation, T cells were stained with LIVE/DEAD Fixable Yellow Dead Cell Stain Kit according to the manufacturer’s instructions (Thermo Fisher Scientific, USA). Cells were then washed and labeled with a combination of antibodies to surface molecules: CD4, CD25, PD-1, PD-L1, and CTLA-4. FOXP3 staining was performed with Foxp3/Transcription Factor Staining Buffer Set (eBioscience) according to the manufacturer’s instruction. Stained cells were acquired on 5-lasers LSRFortessa X20 (BD Bioscience) and analyzed using Flowjo (version 9.7.5 for Mac and 10.6.2 for Windows). The list of all labeled antibodies used in this study can be found in **S2 Table**.

### Mass cytometry (CyTOF)

We designed a panel of antibodies based on surface markers and transcription factors. Antibodies were tagged with a rare metal isotope (**S3 Table**), while for live and dead staining, cisplatin has been used. One million events per samples were acquired on The CyTOF-2 mass cytometer (Fluidigm, USA). Data were normalized based on normalization beads (Ce140, Eu151, Eu153, Ho165, and Lu175). After normalization, FCS files were analyzed using Cytobank platform where automated clustering using t-SNE algorithm was performed on manual gated CD4 (1 × 10^5^ cells per condition) from 3 independent donors. To partition the cells into 50 distinct metaclusters, we applied the FlowSOM clustering algorithm [63]. MEM scores were calculated by using RStudio 3.6.1 following the algorithm described by Diggins and colleagues [64]. FCS files are available on https://flowrepository.org/id/FR-FCM-Z3GZ. Heatmaps were generated using the online tool available on https://software.broadinstitute.org/morpheus/.

### Proliferation and suppression assay

For proliferation assay, CD4^+^CD25^−^ T cells were labeled using Cell Trace Violet (CTV) proliferation kit (Thermo Fisher Scientific) according to the manufacturer’s instructions and stimulated under the different conditions described above in a 96 U-bottom well plate (VWR) at the density of 2 × 10^5^ cells. After stimulation, cells were stained for CD4, CD25, and FOXP3 and analyzed with FACSCanto II flow cytometer (BD). Cell proliferation was determined by monitoring CTV dilution.

For suppression assay, CD4^+^CD25^−^ T cells (Teffs) were labeled with 2.5 μM CFSE for 4 min at room temperature and activated with anti-CD3/CD28 beads (Invitrogen, USA) at 40:1 (cell/bead) ratio. Teffs were then cultured alone (1 × 10^5^) or cocultured with HLA-A2 mismatched iTreg cells at different ratios in a 96 U-bottom well plate. After 5 days, cells were stained with anti-HLA-A2 and acquired on FACSCanto flow cytometer (BD). The percentage of suppression was calculated based on the proliferation of Teffs alone compared with the percentage of proliferation observed in the presence of iTreg cells.

### Phosphoflow cytometry

Flow cytometry to assess protein phosphorylation was performed with True-Phos Perm Buffer (BioLegend) according to the manufacturer’s instruction. Cells were stimulated with 5 μg/mL plate-bound anti-CD3 Ab for 18 h and then with anti-CD3 Ab alone, or in combination with 10 μg/mL plate-bound anti-CD28 Ab or 10 μg/mL plate-bound anti-PD-L1 Ab for 48 h. Briefly, cells were fixed for 15 min at 37°C before permeabilization by slowly adding True-Phos Perm Buffer. Cells were then stained with anti-pSTAT5, anti-pSTAT3, anti-pERK1/2, and anti-pAKT Abs. Stained cells were acquired on LSRFortessa X20 and analyzed using Flowjo.

### Western blotting

Cells were stimulated with 5 μg/mL plate-bound anti-CD3 Ab for 18 h and then with anti-CD3 alone, or in combination with 10 μg/mL plate-bound anti-CD28 Ab or 10 μg/mL plate-bound anti-PD-L1 Ab for 48 h. Cells were then washed with cold PBS and lysed in RIPA buffer (Thermo Fisher Scientific) containing protease inhibitor cocktail (Calbiochem, Germany) for 30 min on ice and centrifuged for 15 min at 15,000*g*. Protein concentrations were determined by Quick Start Bradford assay kit (Bio-Rad, USA), according to the manufacturer’s instructions. Protein lysates were denatured by adding 2X Laemmli buffer (Bio-Rad) containing 5% 2-mercaptoethanol (Sigma, Germany) at 95°C for 5 min. Protein samples were separated on 10% sodium dodecyl sulphate-polyacrylamide gels and transferred onto polyvinylidene difluoride (PVDF) membranes (Millipore, Germany) and probed using anti-phospho-p44/42 MAPK (ERK1/2), anti-p44/42 MAPK (ERK1/2), anti-phospho-S6, anti-S6, and anti-αTubulin, all purchased from Cell Signaling Technology (USA). Detection of the immunoreactive bands was performed with anti-rabbit or anti-mouse HRP-linked antibody (eBioscience) using the ECL Western Blotting Substrate (Bio-Rad). Chemiluminescence was detected with the ImageQuant LASS4000 mini (GE Healthcare Life Science, UK). Blots were quantified using ImageJ Software v1.51k for Mac.

### CRISPR/Cas9-PD1 editing

#### Preparation of crRNA-tracrRNA duplex

The crRNA-tracrRNA duplexes were prepared immediately before experiments by incubating 200 μM of Alt-R CRISPR-Cas9 tracrRNA (IDT) with either the Alt-R CRISPR-Cas9 crRNA targeting PDCD1 (target sequence: GGGCGGTGCTACAACTGGGC) or the Alt-R CRISPR-Cas9 Negative Control crRNA #1 (IDT) at a 1:1 ratio in Nuclease-Free Duplex Buffer (IDT, Cat# 11-01-03-01) at 95°C for 5 min, and then the mix was slowly cooled to room temperature. Nuclease-Free Duplex Buffer was added to the crRNA-tracrRNA duplexes to a final concentration of 61 μM.

#### Cas9 RNP assembly and electroporation

Cas9 RNPs were prepared immediately before experiments by incubating the crRNA-tracrRNA duplex with the Alt-R S.p. Cas9 Nuclease V3 (IDT) in a 2:1 ratio (2.5 μM Cas9 + 5 μM crRNA-tracrRNA duplex per electroporation) for 20 min at room temperature. For each electroporation, the cells were resuspended in PBS and washed at 200*g* for 10 min at room temperature. Per electroporation, 10 million cells were resuspended in 87 μl of P3 primary cell nucleofection solution (Lonza) and mixed with 13.5 μl of Cas9 RNP. Cells with the Cas9 RNP were then transferred to the 100 μL Nucleocuvette Vessel (P3 Primary Cell 4D-Nucleofector X Kit L, Lonza) and electroporated using a 4D nucleofector (Lonza) with program EH-115. After nucleofection, cells were left in 100 μL Nucleocuvette Vessel for 10 min at room temperature. Cells were then transferred to a 6-well plate with X-VIVO15 + 5% human AB serum and incubated at 37°C for 4 to 5 h before reactivation.

### Statistics

Statistical tests were prepared using GraphPad Prism software v8.3. A RM one-way ANOVA and two-way ANOVA were used to compare one related variable between different activations. Post hoc tests were used as indicated in the figure legends. *P* values are reported as follows: *P < 0.05, **P < 0.01, ***P < 0.001, and ****P < 0.0001.

## Supporting information

S1 Fig PD-L1 engagement on CD4^+^CD25^−^ T cells: Evaluation of CTLA-4 surface expression and total cell number. (**A**) Representative counter plots showing CD25 and CTLA-4 surface expression after 72 h of culture of CD4^+^CD25^−^ T cells under the conditions indicated (left panel) and cumulative results expressed as percentage of CTLA-4^+^ T cells (right panel). Data are pooled from at least 3 independent experiments (*n =* 6 different donors). Data are represented using boxplots indicating the min and max and median; **P < 0.01 and ***P < 0.001 by RM one-way ANOVA followed by Tukey multiple comparison. (**B**) Representative counter plots showing the expression of CD25 vs FOXP3 (upper panels) and CD25 vs CTLA-4 (bottom panels) after stimulating CD4^+^CD25^−^ T cells for 72 h with different concentrations of αCD3 and αPD-L1 as indicated. (**C**) Absolute cell number following 72 h of culture under the indicated conditions (*n* = 7 different donors). Data are represented using boxplots indicating the min and max and median. (**D**) Frequency of CD25^high^FOXP3^high^ after stimulating CD4^+^CD25^−^ T cells with αCD3, αCD3/αCD28, and αCD3/αPD-L1 for 72 h. The graph includes data used in Figs 1D and 3 new donors simulated with all antibodies combinations and the αCD3/αPD-L1 combination using 3 extra αPD-L1 antibodies clones: 29E.2A3 (Biolegend), biosimilar Atezolizumab (clone Hu124, R&D), and Durvalumab (clone Hu125, R&D). Bars represent the mean ± SD. Values for each data point can be found in S1 Data. Full gating strategies from representative plots are shown in S1 Gating Strategy. CTLA-4, cytotoxic T-lymphocyte–associated antigen 4; PD-L1, PD-1 ligand 1; RM, repeated measures.
(TIF)

S2 Fig PD-1 CRISPR/Cas9 gene-editing efficiency in primary human CD4^+^ T cells. Cumulative results expressed as percentage of PD-1+ T cells. Data are expressed as mean ± SD and are pooled from at least 2 independent experiments (*n* = 3 different donors). *P < 0.05, **P < 0.01, and ***P < 0.001 were considered significant using two-way ANOVA followed by Tukey multiple comparison test. Data are represented using bars indicating the mean ± SD. Values for each data point can be found in S1 Data. Full gating strategies from representative plots are shown in S1 Gating Strategy. PD-1, Programmed cell death protein 1.
(TIF)

S3 Fig Marker enrichment modeling analysis on total CD4^+^ cells. Heatmap of manually gated live CD4^+^ T cells showing the MEM scores between the different conditions. MEM scores for each condition were generated by using the other 2 populations as reference. Values were mapped from −10 to +10 according to their relative enrichment. MEM, marker enrichment modeling.
(TIF)

S4 Fig High-dimensional data analysis in live CD3^+^ CD8^−^CD4^+^ T cells. **(A)** Heatmap of the aggregate metaclusters showing the median expression of 30 markers. The color in the heatmap represents the median of 0 to 1 scaled expression values of arcsinh transformed data for each marker. The dendrogram shows clustering of samples based on hierarchical clustering with one minus Pearson correlation. Cumulative data showing the percentages of the 50 FlowSOM metaclusters in the CD45^+^CD3^+^CD8^−^CD4^+^ cells. **(B)** Representative map showing the 50 FlowSOM metaclusters from CD4^+^CD25^−^ activated with αCD3, αCD3/αCD28, and αCD3/αPD-L1. Analysis executed on CD45^+^CD3^+^CD8^−^CD4^+^live cells. Cumulative data showing the percentages, on CD45^+^CD3^+^CD8^−^CD4^+^ live cells, of all metaclusters representing **(C)** CD25^−^, **(D)** CD25^+^, **(E)** CD25^+^Ki67^+^, and **(F)** CD25^+^Ki67^−^ cells. Data are expressed as mean ± SD; *P < 0.05 by one-way ANOVA followed by Tukey multiple comparison. Values for each data point can be found in S1 Data.
(TIF)

S5 Fig High-dimensional data analysis in CD4^+^CD25^high^FOXP3^+^ T cells. Cumulative data showing the percentage, on CD45^+^CD3^+^CD8^−^CD4^+^ live cells, of all metaclusters representing **(A)** CD25^high^FOXP3^+^, **(B)** Ki67^+^CD25^high^FOXP3^+^, and **(C)** Ki67^−^CD25^high^FOXP3^+^. Data are expressed as mean ± SD; *P < 0.05 and **P < 0.01 by one-way ANOVA followed by Tukey multiple comparison. **(D)** Heatmap (upper left panel) showing the median expression of 30 markers of the aggregate metaclusters representing CD25^high^FOXP3^+^ cells. The color in the heatmap represents the median of 0 to 1 scaled expression values of arcsinh transformed data for each marker. Stacked bars show the frequency of the 9 CD25^high^FOXP3^+^ clusters for all stimulatory conditions (upper right panel). Representative viSNE maps of manually gated CD45^+^CD3^+^CD8^−^CD4^+^ T cells clustered using surface and intracellular markers. Shown are maps for expression of indicated markers and the CD25^high^FOXP3^+^ metaclusters from FlowSOM analysis. Each colored square represents the 9 different metaclusters. Values for each data point can be found in S1 Data.
(TIF)

S6 Fig High-dimensional data analysis in CD4^+^CD25^High^FOXP3^high^T cells. **(A)** Cumulative data showing the percentage, on CD45^+^CD3^+^CD8^−^CD4^+^ live cells, of all metaclusters representing CD25^high^FOXP3^high^ cells; *P* < 0.05 by RM one-way ANOVA followed by Tukey multiple comparison. (**B**) Representative histograms of CD4^+^CD25^−^ cells, activated using αCD3/αPD-L1, showing the expression of indicated markers in metaclusters 24, 38, 44, and 45. (**C**) Heatmap of CD4^+^CD25^−^ cells, activated using αCD3/αPD-L1, showing the MEM scores between metaclusters 24, 38, 44, and 45. Values for each data point can be found in S1 Data. MEM, marker enrichment modeling; RM, repeated measures.
(TIF)

S7 Fig Kinetics of PD-1 and PD-L1 expression following CD3 and CD3/CD28 stimulation. Representative dot plots showing PD-1 and PD-L1 surface expression on naïve (left panel) and memory (right panel) T cells activated with αCD3 or αCD3/αCD28 for the time indicated. Full gating strategies from representative plots are shown in S1 Gating Strategy. PD-1, Programmed cell death protein 1; PD-L1, PD-1 ligand 1.
(TIF)

S8 Fig Suppressive ability of HD and RA CD4^+^CD25^+^ T cells upon PD-L1 engagement. (**A**) Suppression of CD4^+^CD25^+^FOXP3^+^ T cells from HD and RA following PD-L1 engagement at 1:20 ratio. (**B**) Representative histograms showing CFSE dilution of effector CD4^+^ T cells (1 × 10^5^) activated with αCD3/αCD28 beads at 40:1 (cell/bead) ratio and cultured alone or in the presence of CTV-labeled CD4^+^CD25^+^FOXP3^+^ T cells from HD and RA following PD-L1 engagement cells for 5 days. Values for each data point can be found in S1 Data. Full gating strategies from representative plots are shown in S1 Gating Strategy. CFSE, Carboxyfluorescein succinimidyl ester; CTV, Cell Trace Violet; HD, healthy donor; PD-L1, PD-1 ligand 1; RA, rheumatoid arthritis.
(TIF)

S1 Table Demographic and clinical features of the RA patients included in the study. CCP, cyclic citrullinated peptides; csDMARDs, conventional synthetic Disease Modifying Anti-Rheumatic Drugs; n, number; RA, Rheumatoid Arthritis; RF, Rheumatoid Factor; SD, Standard Deviation. ^1^csDMARDs include Methotrexate, Sulfasalazine, Hydroxychloroquine, either alone or in combination. ^2^Biologics includes certolizumab-pegol (anti-TNFα).
(DOCX)

S2 Table List of antibodies used for flow cytometry. List of antibodies used in this study including clones, fluorochromes, and suppliers.
(DOCX)

S3 Table List of antibodies used for mass cytometry. List of antibodies used in this study including clones, metalTag, and suppliers.
(DOCX)

S1 Data Excel spreadsheet containing, in separate sheets, the numerical data for all figure panels. (XLSX)

S1 Gating Strategy Full gating strategy from representative plots shown in Figs 1A, 1C, 1E, 2A, 2B, 2F, 3A, 3C, 3D, 4A, 5D, 6A, 6E and 7A. (PDF)

S1 Raw Images Raw data images of blots shown in main Figs 5A and 6C and 6D. Blots are cut according to protein markers to allow analysis of multiple proteins from single cell lysates. “X” indicates irrelevant samples or irrelevant membranes.
(PDF)

## Abbreviations

APC: antigen-presenting cell
CTLA-4: cytotoxic T-lymphocyte–associated antigen 4
CyTOF: cytometry by time-of-flight
IFN: interferon
iTreg: inducible Treg
MAPK: mitogen-activated protein kinase
MEM: marker enrichment modeling
PD-1: Programmed cell death protein 1
PD-L1: PD-1 ligand
PEAC: Pathobiology of Early Arthritis Cohort
pS6: phosphorylated S6
PVDF: polyvinylidene difluoride
RA: rheumatoid arthritis
TCR: T cell receptor
Treg: regulatory T cell

## References

[1] Long SA, Buckner JH. CD4+FOXP3+ T regulatory cells in human autoimmunity: more than a numbers game. J Immunol. 2011;187(5):2061–6. Epub 2011/08/23. 10.4049/jimmunol.1003224 21856944 PMC3160735

[2] Romano M, Tung SL, Smyth LA, Lombardi G. Treg therapy in transplantation: a general overview. Transpl Int. 2017;30(8):745–53. Epub 2016/12/25. 10.1111/tri.12909 . 28012226

[3] Romano M, Fanelli G, Albany CJ, Giganti G, Lombardi G. Past, Present, and Future of Regulatory T Cell Therapy in Transplantation and Autoimmunity. Front Immunol. 2019;10:43. Epub 2019/02/26. 10.3389/fimmu.2019.00043 30804926 PMC6371029

[4] Miyao T, Floess S, Setoguchi R, Luche H, Fehling HJ, Waldmann H, et al. Plasticity of Foxp3(+) T cells reflects promiscuous Foxp3 expression in conventional T cells but not reprogramming of regulatory T cells. Immunity. 2012;36(2):262–75. Epub 2012/02/14. 10.1016/j.immuni.2011.12.012 . 22326580

[5] Sakaguchi S, Miyara M, Costantino CM, Hafler DA. FOXP3+ regulatory T cells in the human immune system. Nat Rev Immunol. 2010;10(7):490–500. Epub 2010/06/19. 10.1038/nri2785 . 20559327

[6] Schmitt EG, Williams CB. Generation and function of induced regulatory T cells. Front Immunol. 2013;4:152. Epub 2013/06/27. 10.3389/fimmu.2013.00152 23801990 PMC3685796

[7] Hou TZ, Qureshi OS, Wang CJ, Baker J, Young SP, Walker LS, et al. A transendocytosis model of CTLA-4 function predicts its suppressive behavior on regulatory T cells. J Immunol. 2015;194(5):2148–59. Epub 2015/01/30. 10.4049/jimmunol.1401876 25632005 PMC4522736

[8] Fife BT, Pauken KE, Eagar TN, Obu T, Wu J, Tang Q, et al. Interactions between PD-1 and PD-L1 promote tolerance by blocking the TCR-induced stop signal. Nat Immunol. 2009;10(11):1185–92. Epub 2009/09/29. 10.1038/ni.1790 19783989 PMC2778301

[9] Schneider H, Downey J, Smith A, Zinselmeyer BH, Rush C, Brewer JM, et al. Reversal of the TCR stop signal by CTLA-4. Science. 2006;313(5795):1972–5. Epub 2006/08/26. 10.1126/science.1131078 . 16931720

[10] Francisco LM, Salinas VH, Brown KE, Vanguri VK, Freeman GJ, Kuchroo VK, et al. PD-L1 regulates the development, maintenance, and function of induced regulatory T cells. J Exp Med. 2009;206(13):3015–29. Epub 2009/12/17. 10.1084/jem.20090847 20008522 PMC2806460

[11] Amarnath S, Mangus CW, Wang JC, Wei F, He A, Kapoor V, et al. The PDL1-PD1 axis converts human TH1 cells into regulatory T cells. Sci Transl Med. 2011;3(111):111ra20. Epub 2011/12/03. 10.1126/scitranslmed.3003130 22133721 PMC3235958

[12] Fife BT, Bluestone JA. Control of peripheral T-cell tolerance and autoimmunity via the CTLA-4 and PD-1 pathways. Immunol Rev. 2008;224:166–82. Epub 2008/09/02. 10.1111/j.1600-065X.2008.00662.x . 18759926

[13] Hino R, Kabashima K, Kato Y, Yagi H, Nakamura M, Honjo T, et al. Tumor cell expression of programmed cell death-1 ligand 1 is a prognostic factor for malignant melanoma. Cancer. 2010;116(7):1757–66. Epub 2010/02/10. 10.1002/cncr.24899 . 20143437

[14] Wei F, Zhong S, Ma Z, Kong H, Medvec A, Ahmed R, et al. Strength of PD-1 signaling differentially affects T-cell effector functions. Proc Natl Acad Sci U S A. 2013;110(27):E2480–9. Epub 2013/04/24. 10.1073/pnas.1305394110 23610399 PMC3703988

[15] Azuma T, Yao S, Zhu G, Flies AS, Flies SJ, Chen L. B7-H1 is a ubiquitous antiapoptotic receptor on cancer cells. Blood. 2008;111(7):3635–43. Epub 2008/01/29. 10.1182/blood-2007-11-123141 18223165 PMC2275025

[16] Gato-Canas M, Zuazo M, Arasanz H, Ibanez-Vea M, Lorenzo L, Fernandez-Hinojal G, et al. PDL1 Signals through Conserved Sequence Motifs to Overcome Interferon-Mediated Cytotoxicity. Cell Rep. 2017;20(8):1818–29. Epub 2017/08/24. 10.1016/j.celrep.2017.07.075 . 28834746

[17] Diskin B, Adam S, Cassini MF, Sanchez G, Liria M, Aykut B, et al. PD-L1 engagement on T cells promotes self-tolerance and suppression of neighboring macrophages and effector T cells in cancer. Nat Immunol. 2020. Epub 2020/03/11. 10.1038/s41590-020-0620-x . 32152508

[18] Dai S, Jia R, Zhang X, Fang Q, Huang L. The PD-1/PD-Ls pathway and autoimmune diseases. Cell Immunol. 2014;290(1):72–9. Epub 2014/06/09. 10.1016/j.cellimm.2014.05.006 . 24908630

[19] Gottschalk RA, Corse E, Allison JP. TCR ligand density and affinity determine peripheral induction of Foxp3 in vivo. J Exp Med. 2010;207(8):1701–11. Epub 2010/07/28. 10.1084/jem.20091999 20660617 PMC2916126

[20] Scotta C, Soligo M, Camperio C, Piccolella E. FOXP3 induced by CD28/B7 interaction regulates CD25 and anergic phenotype in human CD4+CD25- T lymphocytes. J Immunol. 2008;181(2):1025–33. Epub 2008/07/09. 10.4049/jimmunol.181.2.1025 . 18606654

[21] Zheng SG, Wang J, Wang P, Gray JD, Horwitz DA. IL-2 is essential for TGF-beta to convert naive CD4+CD25- cells to CD25+Foxp3+ regulatory T cells and for expansion of these cells. J Immunol. 2007;178(4):2018–27. Epub 2007/02/06. 10.4049/jimmunol.178.4.2018 . 17277105

[22] Long SA, Rieck M, Tatum M, Bollyky PL, Wu RP, Muller I, et al. Low-dose antigen promotes induction of FOXP3 in human CD4+ T cells. J Immunol. 2011;187(7):3511–20. Epub 2011/08/26. 10.4049/jimmunol.1003880 21865550 PMC3178710

[23] Tran DQ, Ramsey H, Shevach EM. Induction of FOXP3 expression in naive human CD4+FOXP3 T cells by T-cell receptor stimulation is transforming growth factor-beta dependent but does not confer a regulatory phenotype. Blood. 2007;110(8):2983–90. Epub 2007/07/24. 10.1182/blood-2007-06-094656 17644734 PMC2018674

[24] Kinter AL, Godbout EJ, McNally JP, Sereti I, Roby GA, O’Shea MA, et al. The common gamma-chain cytokines IL-2, IL-7, IL-15, and IL-21 induce the expression of programmed death-1 and its ligands. J Immunol. 2008;181(10):6738–46. Epub 2008/11/05. 10.4049/jimmunol.181.10.6738 . 18981091

[25] Chaudhry A, Samstein RM, Treuting P, Liang Y, Pils MC, Heinrich JM, et al. Interleukin-10 signaling in regulatory T cells is required for suppression of Th17 cell-mediated inflammation. Immunity. 2011;34(4):566–78. Epub 2011/04/23. 10.1016/j.immuni.2011.03.018 21511185 PMC3088485

[26] Laidlaw BJ, Cui W, Amezquita RA, Gray SM, Guan T, Lu Y, et al. Production of IL-10 by CD4(+) regulatory T cells during the resolution of infection promotes the maturation of memory CD8(+) T cells. Nat Immunol. 2015;16(8):871–9. Epub 2015/07/07. 10.1038/ni.3224 26147684 PMC4713030

[27] Scotta C, Esposito M, Fazekasova H, Fanelli G, Edozie FC, Ali N, et al. Differential effects of rapamycin and retinoic acid on expansion, stability and suppressive qualities of human CD4(+)CD25(+)FOXP3(+) T regulatory cell subpopulations. Haematologica. 2013;98(8):1291–9. Epub 2012/12/18. 10.3324/haematol.2012.074088 23242600 PMC3729911

[28] Hester J, Schiopu A, Nadig SN, Wood KJ. Low-dose rapamycin treatment increases the ability of human regulatory T cells to inhibit transplant arteriosclerosis in vivo. Am J Transplant. 2012;12(8):2008–16. Epub 2012/04/17. 10.1111/j.1600-6143.2012.04065.x 22500984 PMC3440570

[29] Sun IH, Oh MH, Zhao L, Patel CH, Arwood ML, Xu W, et al. mTOR Complex 1 Signaling Regulates the Generation and Function of Central and Effector Foxp3(+) Regulatory T Cells. J Immunol. 2018;201(2):481–92. Epub 2018/06/10. 10.4049/jimmunol.1701477 29884702 PMC6089237

[30] Passerini L, Allan SE, Battaglia M, Di Nunzio S, Alstad AN, Levings MK, et al. STAT5-signaling cytokines regulate the expression of FOXP3 in CD4+CD25+ regulatory T cells and CD4+CD25- effector T cells. Int Immunol. 2008;20(3):421–31. Epub 2008/02/14. 10.1093/intimm/dxn002 . 18270368

[31] Chaudhry A, Rudra D, Treuting P, Samstein RM, Liang Y, Kas A, et al. CD4+ regulatory T cells control TH17 responses in a Stat3-dependent manner. Science. 2009;326(5955):986–91. Epub 2009/10/03. 10.1126/science.1172702 19797626 PMC4408196

[32] Singh K, Deshpande P, Pryshchep S, Colmegna I, Liarski V, Weyand CM, et al. ERK-dependent T cell receptor threshold calibration in rheumatoid arthritis. J Immunol. 2009;183(12):8258–67. Epub 2009/12/17. 10.4049/jimmunol.0901784 20007589 PMC2828269

[33] Singh K, Deshpande P, Li G, Yu M, Pryshchep S, Cavanagh M, et al. K-RAS GTPase- and B-RAF kinase-mediated T-cell tolerance defects in rheumatoid arthritis. Proc Natl Acad Sci U S A. 2012;109(25):E1629–37. Epub 2012/05/23. 10.1073/pnas.1117640109 22615393 PMC3382540

[34] Zheng Y, Manzotti CN, Burke F, Dussably L, Qureshi O, Walker LS, et al. Acquisition of suppressive function by activated human CD4+ CD25- T cells is associated with the expression of CTLA-4 not FoxP3. J Immunol. 2008;181(3):1683–91. Epub 2008/07/22. 10.4049/jimmunol.181.3.1683 18641304 PMC2758479

[35] Takeshita M, Suzuki K, Kondo Y, Morita R, Okuzono Y, Koga K, et al. Multi-dimensional analysis identified rheumatoid arthritis-driving pathway in human T cell. Ann Rheum Dis. 2019;78(10):1346–56. Epub 2019/06/07. 10.1136/annrheumdis-2018-214885 31167762 PMC6788883

[36] Alsaab HO, Sau S, Alzhrani R, Tatiparti K, Bhise K, Kashaw SK, et al. PD-1 and PD-L1 Checkpoint Signaling Inhibition for Cancer Immunotherapy: Mechanism, Combinations, and Clinical Outcome. Front Pharmacol. 2017;8:561. Epub 2017/09/08. 10.3389/fphar.2017.00561 28878676 PMC5572324

[37] Cappelli LC, Shah AA, Bingham CO 3rd. Immune-Related Adverse Effects of Cancer Immunotherapy- Implications for Rheumatology. Rheum Dis Clin North Am. 2017;43(1):65–78. Epub 2016/11/29. 10.1016/j.rdc.2016.09.007 27890174 PMC5127444

[38] Swaika A, Hammond WA, Joseph RW. Current state of anti-PD-L1 and anti-PD-1 agents in cancer therapy. Mol Immunol. 2015;67(2 Pt A):4–17. Epub 2015/03/10. 10.1016/j.molimm.2015.02.009 . 25749122

[39] Juneja VR, McGuire KA, Manguso RT, LaFleur MW, Collins N, Haining WN, et al. PD-L1 on tumor cells is sufficient for immune evasion in immunogenic tumors and inhibits CD8 T cell cytotoxicity. J Exp Med. 2017;214(4):895–904. Epub 2017/03/18. 10.1084/jem.20160801 28302645 PMC5379970

[40] Lamb JR, Feldmann M. Essential requirement for major histocompatibility complex recognition in T-cell tolerance induction. Nature. 1984;308(5954):72–4. Epub 1984/03/01. 10.1038/308072a0 . 6199675

[41] Salgado FJ, Lojo J, Fernandez-Alonso CM, Vinuela J, Cordero OJ, Nogueira M. Interleukin-dependent modulation of HLA-DR expression on CD4and CD8 activated T cells. Immunol Cell Biol. 2002;80(2):138–47. Epub 2002/04/10. 10.1046/j.1440-1711.2002.01055.x . 11940114

[42] Baecher-Allan C, Hafler DA. Human regulatory T cells and their role in autoimmune disease. Immunol Rev. 2006;212:203–16. Epub 2006/08/15. 10.1111/j.0105-2896.2006.00417.x . 16903916

[43] Tatari-Calderone Z, Semnani RT, Nutman TB, Schlom J, Sabzevari H. Acquisition of CD80 by human T cells at early stages of activation: functional involvement of CD80 acquisition in T cell to T cell interaction. J Immunol. 2002;169(11):6162–9. Epub 2002/11/22. 10.4049/jimmunol.169.11.6162 . 12444120

[44] Jago CB, Yates J, Camara NO, Lechler RI, Lombardi G. Differential expression of CTLA-4 among T cell subsets. Clin Exp Immunol. 2004;136(3):463–71. Epub 2004/05/19. 10.1111/j.1365-2249.2004.02478.x 15147348 PMC1809051

[45] Perkins D, Wang Z, Donovan C, He H, Mark D, Guan G, et al. Regulation of CTLA-4 expression during T cell activation. J Immunol. 1996;156(11):4154–9. Epub 1996/06/01. . 8666782

[46] Lombardi G, Sidhu S, Batchelor R, Lechler R. Anergic T cells as suppressor cells in vitro. Science. 1994;264(5165):1587–9. Epub 1994/06/10. 10.1126/science.8202711 . 8202711

[47] Schooten E, Klous P, van den Elsen PJ, Holling TM. Lack of MHC-II expression in activated mouse T cells correlates with DNA methylation at the CIITA-PIII region. Immunogenetics. 2005;57(10):795–9. Epub 2005/10/20. 10.1007/s00251-005-0051-8 . 16235089

[48] Raffin C, Pignon P, Celse C, Debien E, Valmori D, Ayyoub M. Human memory Helios- FOXP3+ regulatory T cells (Tregs) encompass induced Tregs that express Aiolos and respond to IL-1beta by downregulating their suppressor functions. J Immunol. 2013;191(9):4619–27. Epub 2013/09/27. 10.4049/jimmunol.1301378 . 24068664

[49] Wang L, Pino-Lagos K, de Vries VC, Guleria I, Sayegh MH, Noelle RJ. Programmed death 1 ligand signaling regulates the generation of adaptive Foxp3+CD4+ regulatory T cells. Proc Natl Acad Sci U S A. 2008;105(27):9331–6. Epub 2008/07/05. 10.1073/pnas.0710441105 18599457 PMC2442817

[50] Keir ME, Butte MJ, Freeman GJ, Sharpe AH. PD-1 and its ligands in tolerance and immunity. Annu Rev Immunol. 2008;26:677–704. Epub 2008/01/05. 10.1146/annurev.immunol.26.021607.090331 . 18173375 PMC10637733

[51] Butte MJ, Keir ME, Phamduy TB, Sharpe AH, Freeman GJ. Programmed death-1 ligand 1 interacts specifically with the B7-1 costimulatory molecule to inhibit T cell responses. Immunity. 2007;27(1):111–22. Epub 2007/07/17. 10.1016/j.immuni.2007.05.016 17629517 PMC2707944

[52] Park JJ, Omiya R, Matsumura Y, Sakoda Y, Kuramasu A, Augustine MM, et al. B7-H1/CD80 interaction is required for the induction and maintenance of peripheral T-cell tolerance. Blood. 2010;116(8):1291–8. Epub 2010/05/18. 10.1182/blood-2010-01-265975 20472828 PMC2938239

[53] Gagliani N, Amezcua Vesely MC, Iseppon A, Brockmann L, Xu H, Palm NW, et al. Th17 cells transdifferentiate into regulatory T cells during resolution of inflammation. Nature. 2015;523(7559):221–5. Epub 2015/04/30. 10.1038/nature14452 25924064 PMC4498984

[54] Vukmanovic-Stejic M, Zhang Y, Cook JE, Fletcher JM, McQuaid A, Masters JE, et al. Human CD4+ CD25hi Foxp3+ regulatory T cells are derived by rapid turnover of memory populations in vivo. J Clin Invest. 2006;116(9):2423–33. Epub 2006/09/07. 10.1172/JCI28941 16955142 PMC1555646

[55] Kretschmer K, Apostolou I, Hawiger D, Khazaie K, Nussenzweig MC, von Boehmer H. Inducing and expanding regulatory T cell populations by foreign antigen. Nat Immunol. 2005;6(12):1219–27. Epub 2005/10/26. 10.1038/ni1265 . 16244650

[56] Sauer S, Bruno L, Hertweck A, Finlay D, Leleu M, Spivakov M, et al. T cell receptor signaling controls Foxp3 expression via PI3K, Akt, and mTOR. Proc Natl Acad Sci U S A. 2008;105(22):7797–802. Epub 2008/05/30. 10.1073/pnas.0800928105 18509048 PMC2409380

[57] Lin JT, Stein EA, Wong MT, Kalpathy KJ, Su LL, Utz PJ, et al. Differential mTOR and ERK pathway utilization by effector CD4 T cells suggests combinatorial drug therapy of arthritis. Clin Immunol. 2012;142(2):127–38. Epub 2011/11/15. 10.1016/j.clim.2011.09.008 22075384 PMC3273543

[58] Li MO, Rudensky AY. T cell receptor signalling in the control of regulatory T cell differentiation and function. Nat Rev Immunol. 2016;16(4):220–33. Epub 2016/03/31. 10.1038/nri.2016.26 27026074 PMC4968889

[59] Hsu P, Santner-Nanan B, Hu M, Skarratt K, Lee CH, Stormon M, et al. IL-10 Potentiates Differentiation of Human Induced Regulatory T Cells via STAT3 and Foxo1. J Immunol. 2015;195(8):3665–74. Epub 2015/09/13. 10.4049/jimmunol.1402898 . 26363058

[60] Afzali B, Mitchell PJ, Edozie FC, Povoleri GA, Dowson SE, Demandt L, et al. CD161 expression characterizes a subpopulation of human regulatory T cells that produces IL-17 in a STAT3-dependent manner. Eur J Immunol. 2013;43(8):2043–54. Epub 2013/05/17. 10.1002/eji.201243296 23677517 PMC3815561

[61] Hossain DM, Panda AK, Manna A, Mohanty S, Bhattacharjee P, Bhattacharyya S, et al. FoxP3 acts as a cotranscription factor with STAT3 in tumor-induced regulatory T cells. Immunity. 2013;39(6):1057–69. Epub 2013/12/10. 10.1016/j.immuni.2013.11.005 . 24315995

[62] Belkhir R, Burel SL, Dunogeant L, Marabelle A, Hollebecque A, Besse B, et al. Rheumatoid arthritis and polymyalgia rheumatica occurring after immune checkpoint inhibitor treatment. Ann Rheum Dis. 2017;76(10):1747–50. Epub 2017/06/11. 10.1136/annrheumdis-2017-211216 . 28600350

[63] Van Gassen S, Callebaut B, Van Helden MJ, Lambrecht BN, Demeester P, Dhaene T, et al. FlowSOM: Using self-organizing maps for visualization and interpretation of cytometry data. Cytometry A. 2015;87(7):636–45. Epub 2015/01/13. 10.1002/cyto.a.22625 . 25573116

[64] Diggins KE, Greenplate AR, Leelatian N, Wogsland CE, Irish JM. Characterizing cell subsets using marker enrichment modeling. Nat Methods. 2017;14(3):275–8. Epub 2017/01/31. 10.1038/nmeth.4149 28135256 PMC5330853

