# Supplementary figures and images for: PD-L1 signaling on human memory CD4+ T cells induces a regulatory phenotype
Source: PLoS Biol. 2021 Apr 26;19(4):e3001199. doi: 10.1371/journal.pbio.3001199 (PMC8101994; doi:10.1371/journal.pbio.3001199)

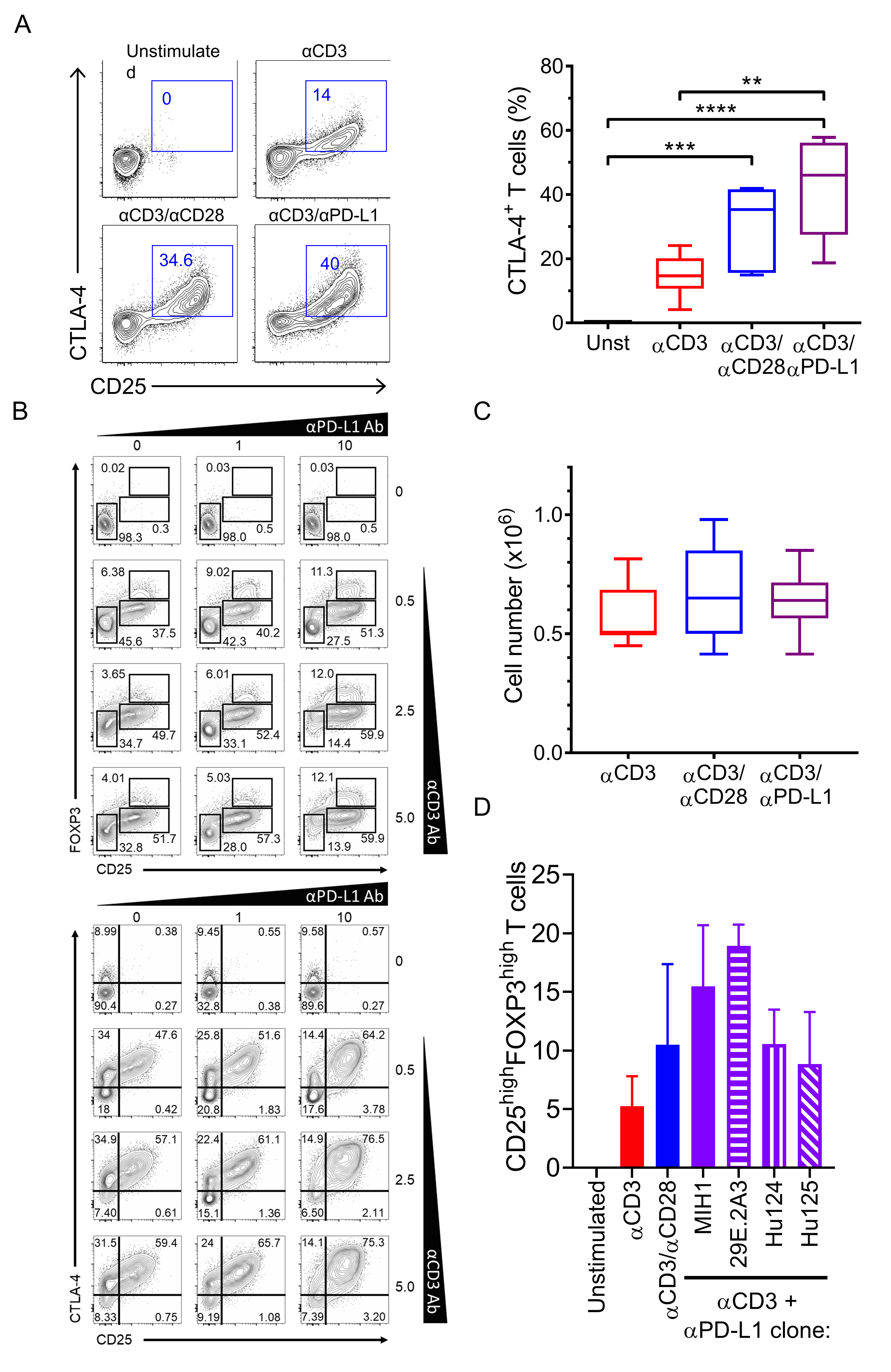

Supplement: S1 Fig — (A) Representative counter plots showing CD25 and CTLA-4 surface expression after 72 h of culture of CD4+CD25− T cells under the conditions indicated (left panel) and cumulative results expressed as percentage of CTLA-4+ T cells (right panel). Data are pooled from at least 3 independent experiments (n = 6 different donors). Data are represented using boxplots indicating the min and max and median; **P < 0.01 and ***P < 0.001 by RM one-way ANOVA followed by Tukey multiple comparison. (B) Representative counter plots showing the expression of CD25 vs FOXP3 (upper panels) and CD25 vs CTLA-4 (bottom panels) after stimulating CD4+CD25− T cells for 72 h with different concentrations of αCD3 and αPD-L1 as indicated. (C) Absolute cell number following 72 h of culture under the indicated conditions (n = 7 different donors). Data are represented using boxplots indicating the min and max and median. (D) Frequency of CD25highFOXP3high after stimulating CD4+CD25− T cells with αCD3, αCD3/αCD28, and αCD3/αPD-L1 for 72 h. The graph includes data used in Figs 1D and 3 new donors simulated with all antibodies combinations and the αCD3/αPD-L1 combination using 3 extra αPD-L1 antibodies clones: 29E.2A3 (Biolegend), biosimilar Atezolizumab (clone Hu124, R&D), and Durvalumab (clone Hu125, R&D). Bars represent the mean ± SD. Values for each data point can be found in S1 Data. Full gating strategies from representative plots are shown in S1 Gating Strategy. CTLA-4, cytotoxic T-lymphocyte–associated antigen 4; PD-L1, PD-1 ligand 1; RM, repeated measures. (TIF) [file pbio.3001199.s001.tif]

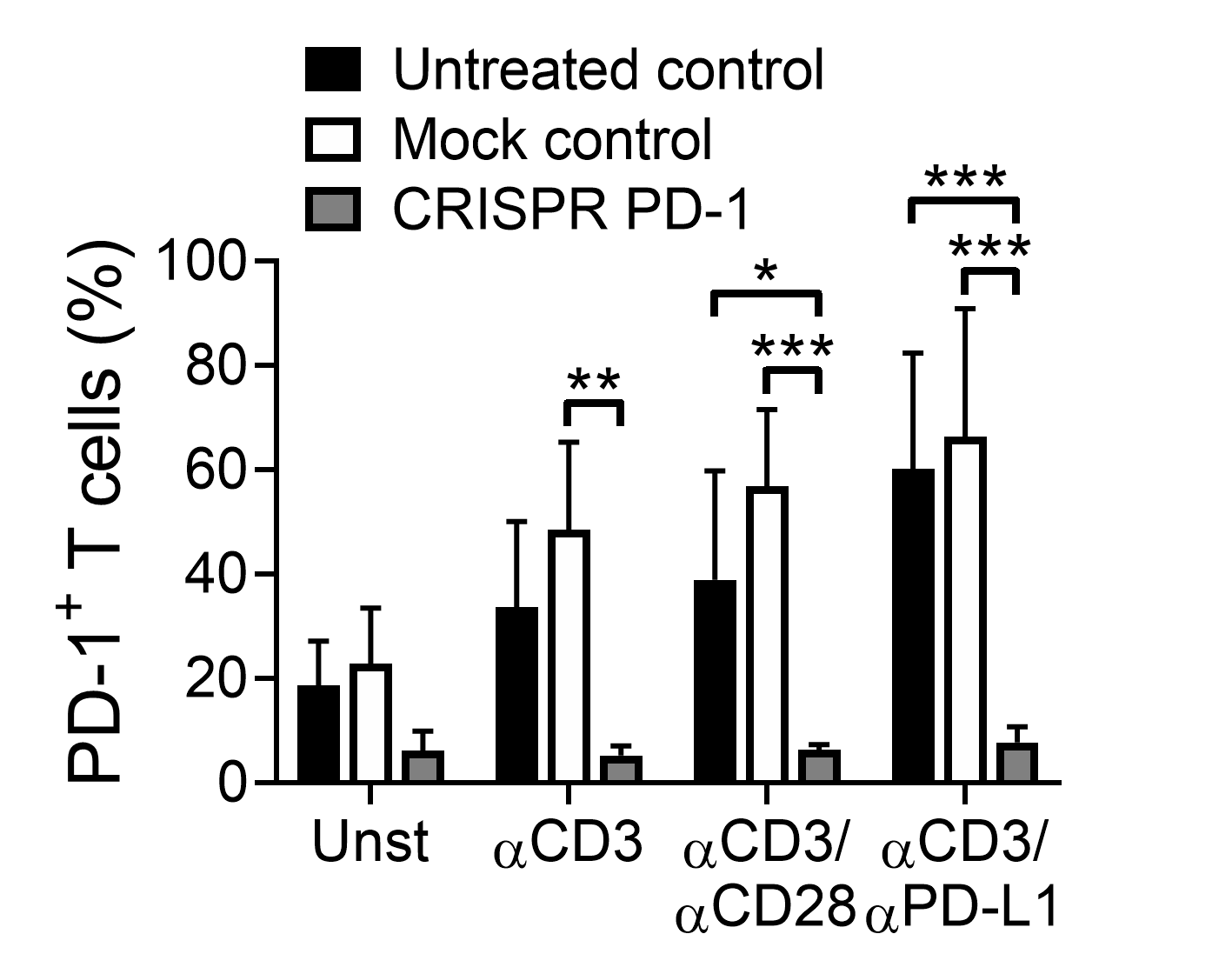

Supplement: S2 Fig — Cumulative results expressed as percentage of PD-1+ T cells. Data are expressed as mean ± SD and are pooled from at least 2 independent experiments (n = 3 different donors). *P < 0.05, **P < 0.01, and ***P < 0.001 were considered significant using two-way ANOVA followed by Tukey multiple comparison test. Data are represented using bars indicating the mean ± SD. Values for each data point can be found in S1 Data. Full gating strategies from representative plots are shown in S1 Gating Strategy. PD-1, Programmed cell death protein 1. (TIF) [file pbio.3001199.s002.tif]

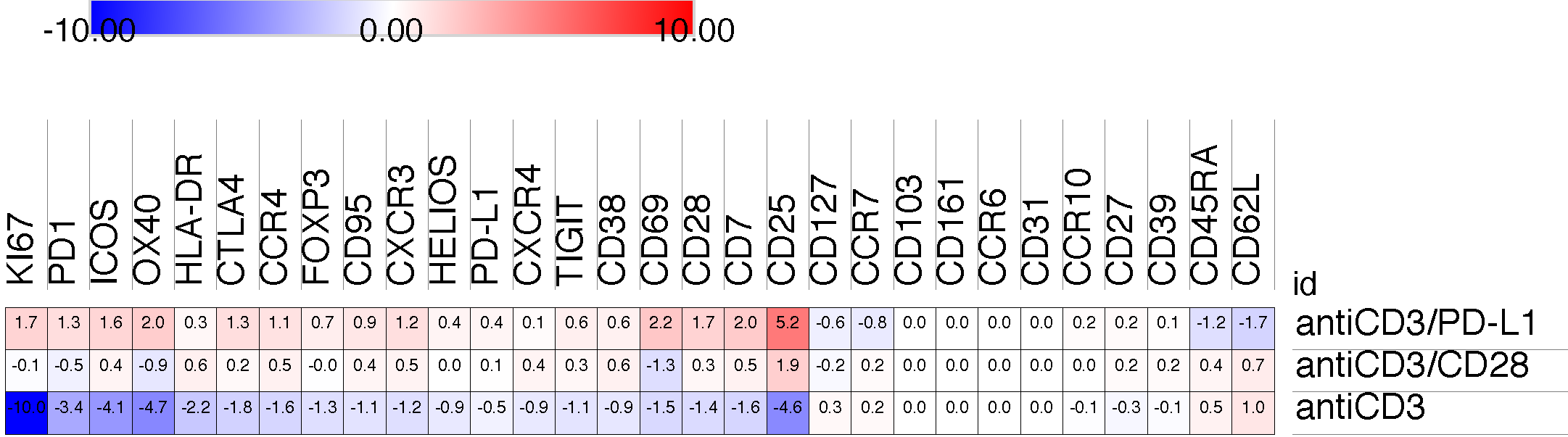

Supplement: S3 Fig — Heatmap of manually gated live CD4+ T cells showing the MEM scores between the different conditions. MEM scores for each condition were generated by using the other 2 populations as reference. Values were mapped from −10 to +10 according to their relative enrichment. MEM, marker enrichment modeling. (TIF) [file pbio.3001199.s003.tif]

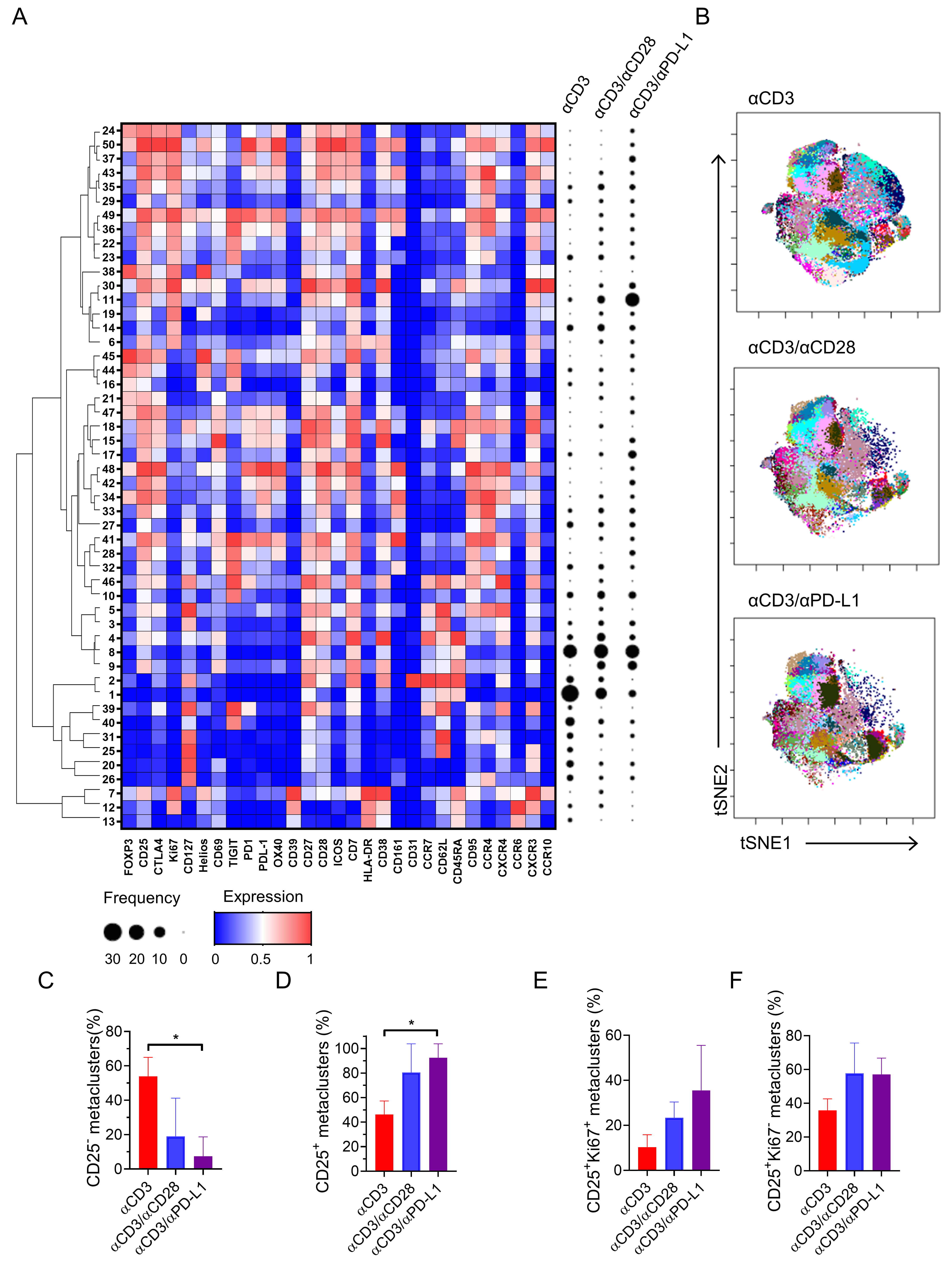

Supplement: S4 Fig — (A) Heatmap of the aggregate metaclusters showing the median expression of 30 markers. The color in the heatmap represents the median of 0 to 1 scaled expression values of arcsinh transformed data for each marker. The dendrogram shows clustering of samples based on hierarchical clustering with one minus Pearson correlation. Cumulative data showing the percentages of the 50 FlowSOM metaclusters in the CD45+CD3+CD8−CD4+ cells. (B) Representative map showing the 50 FlowSOM metaclusters from CD4+CD25− activated with αCD3, αCD3/αCD28, and αCD3/αPD-L1. Analysis executed on CD45+CD3+CD8−CD4+live cells. Cumulative data showing the percentages, on CD45+CD3+CD8−CD4+ live cells, of all metaclusters representing (C) CD25−, (D) CD25+, (E) CD25+Ki67+, and (F) CD25+Ki67− cells. Data are expressed as mean ± SD; *P < 0.05 by one-way ANOVA followed by Tukey multiple comparison. Values for each data point can be found in S1 Data. (TIF) [file pbio.3001199.s004.tif]

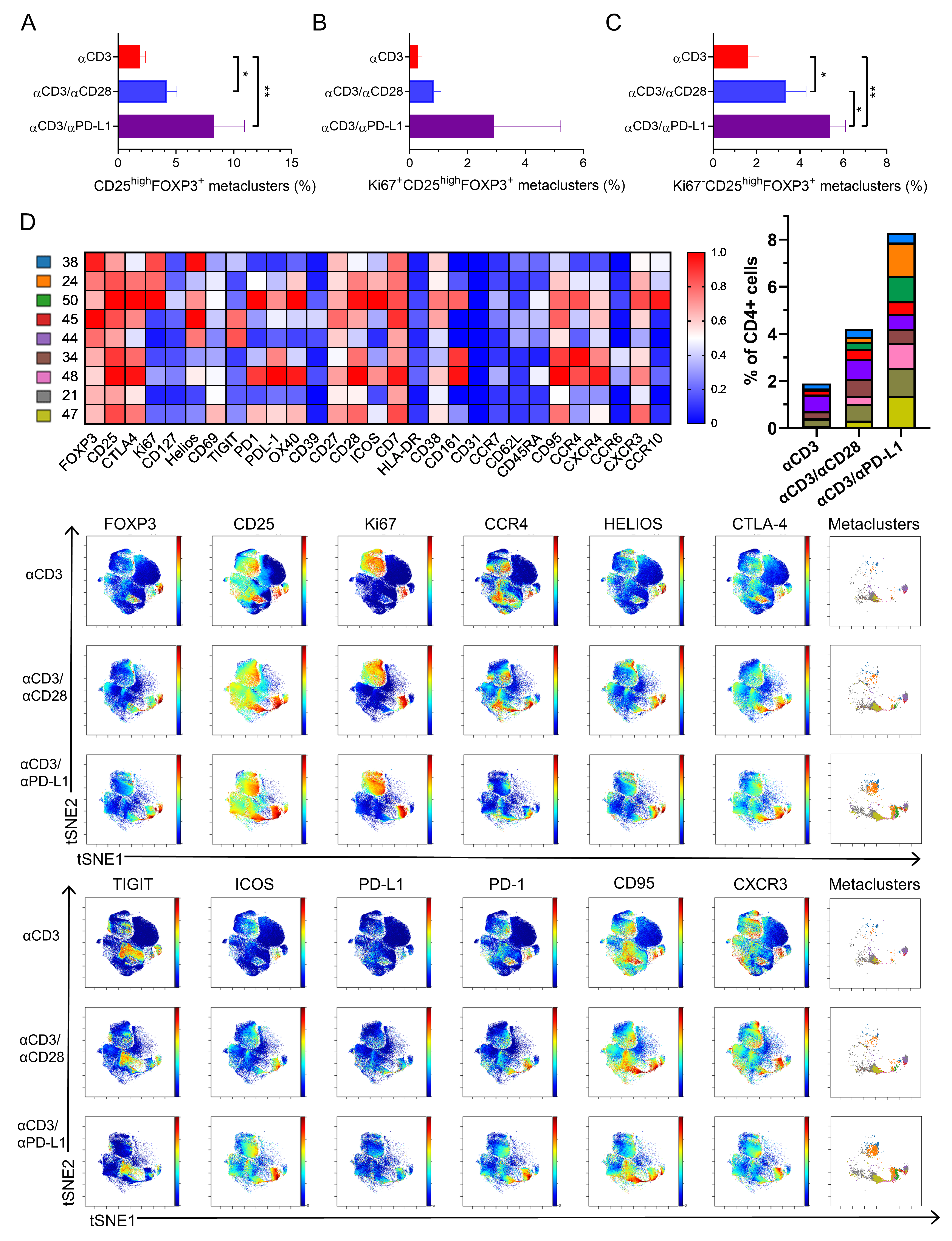

Supplement: S5 Fig — Cumulative data showing the percentage, on CD45+CD3+CD8−CD4+ live cells, of all metaclusters representing (A) CD25highFOXP3+, (B) Ki67+CD25highFOXP3+, and (C) Ki67−CD25highFOXP3+. Data are expressed as mean ± SD; *P < 0.05 and **P < 0.01 by one-way ANOVA followed by Tukey multiple comparison. (D) Heatmap (upper left panel) showing the median expression of 30 markers of the aggregate metaclusters representing CD25highFOXP3+ cells. The color in the heatmap represents the median of 0 to 1 scaled expression values of arcsinh transformed data for each marker. Stacked bars show the frequency of the 9 CD25highFOXP3+ clusters for all stimulatory conditions (upper right panel). Representative viSNE maps of manually gated CD45+CD3+CD8−CD4+ T cells clustered using surface and intracellular markers. Shown are maps for expression of indicated markers and the CD25highFOXP3+ metaclusters from FlowSOM analysis. Each colored square represents the 9 different metaclusters. Values for each data point can be found in S1 Data. (TIF) [file pbio.3001199.s005.tif]

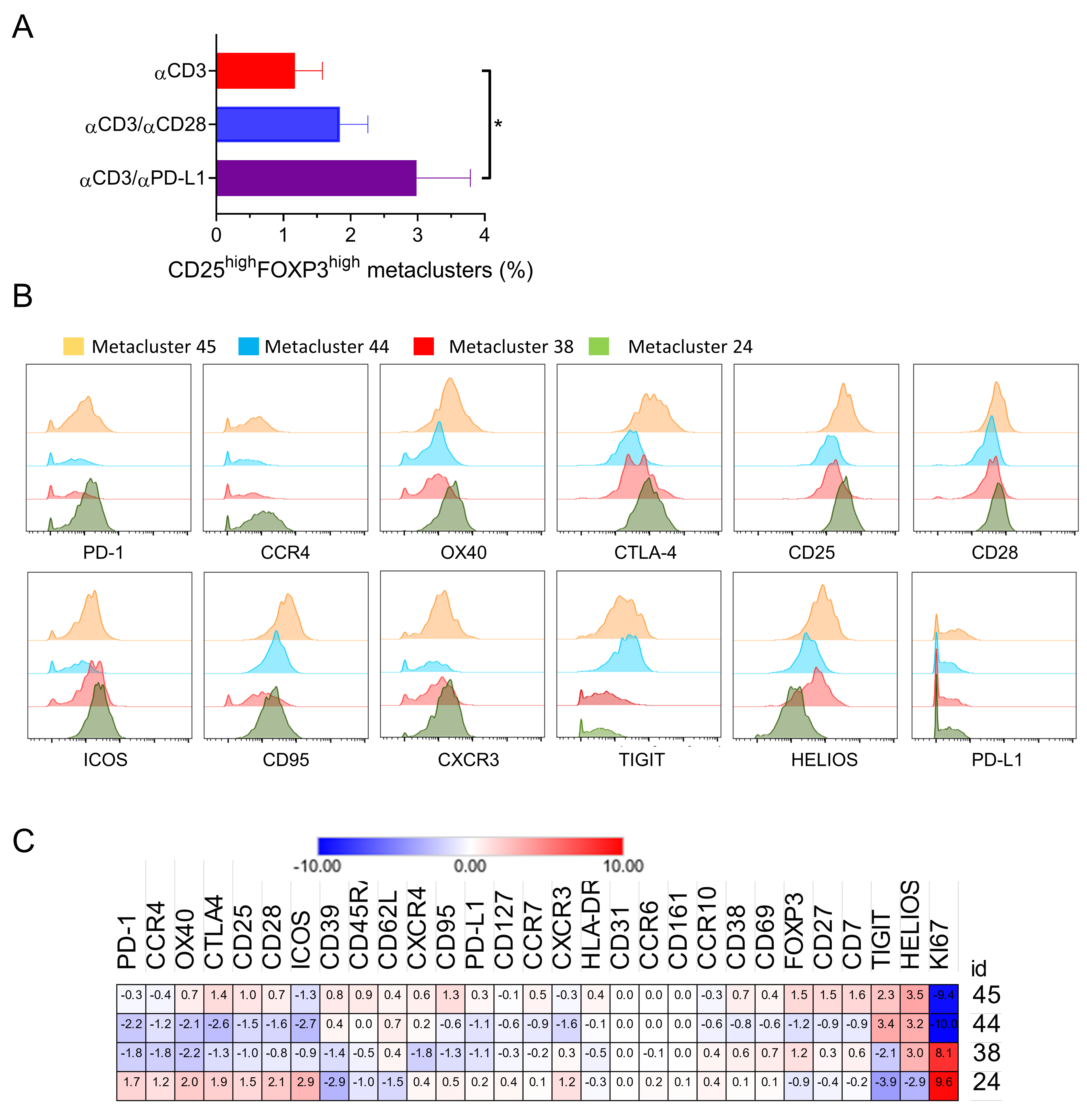

Supplement: S6 Fig — (A) Cumulative data showing the percentage, on CD45+CD3+CD8−CD4+ live cells, of all metaclusters representing CD25highFOXP3high cells; P < 0.05 by RM one-way ANOVA followed by Tukey multiple comparison. (B) Representative histograms of CD4+CD25− cells, activated using αCD3/αPD-L1, showing the expression of indicated markers in metaclusters 24, 38, 44, and 45. (C) Heatmap of CD4+CD25− cells, activated using αCD3/αPD-L1, showing the MEM scores between metaclusters 24, 38, 44, and 45. Values for each data point can be found in S1 Data. MEM, marker enrichment modeling; RM, repeated measures. (TIF) [file pbio.3001199.s006.tif]

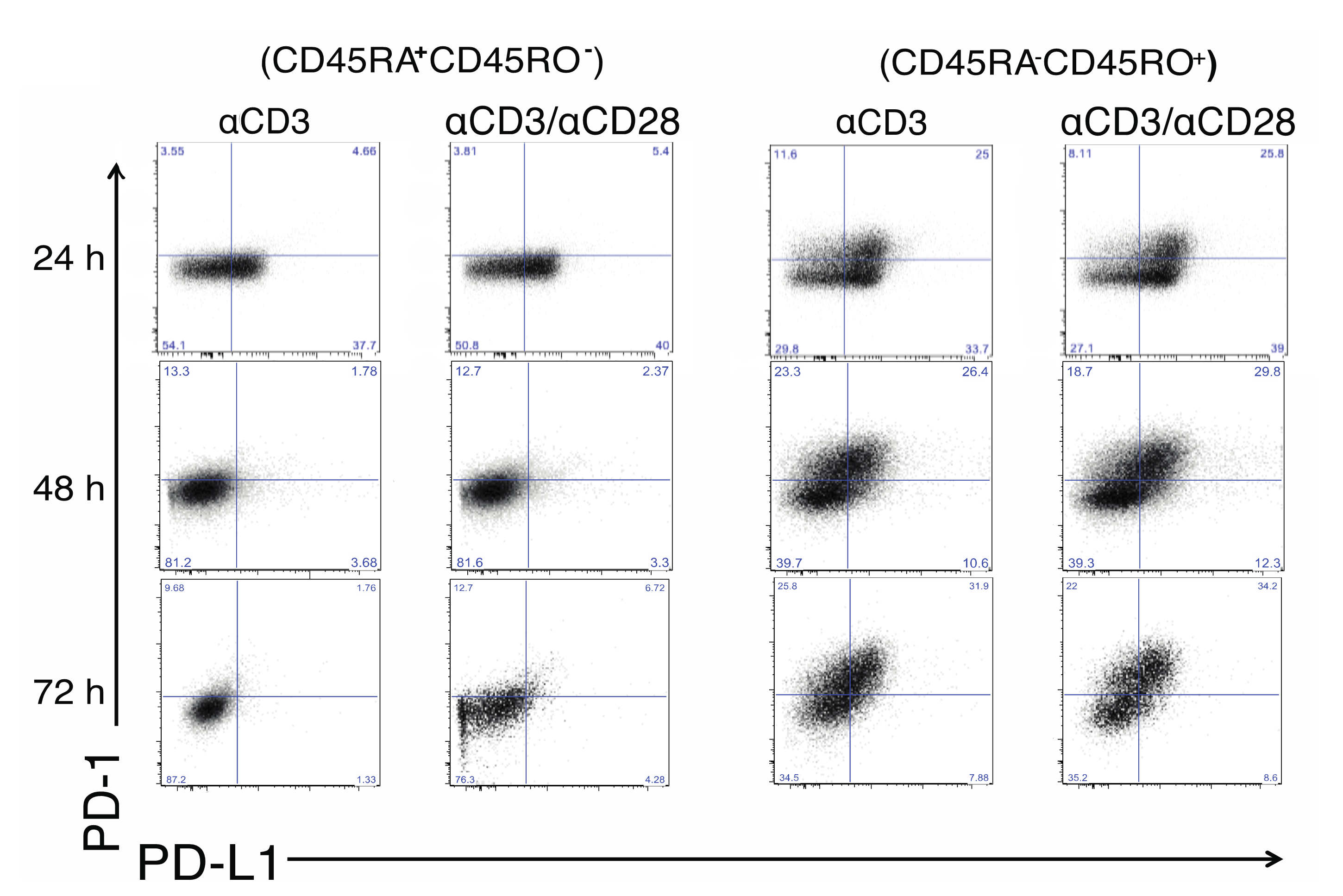

Supplement: S7 Fig — Representative dot plots showing PD-1 and PD-L1 surface expression on naïve (left panel) and memory (right panel) T cells activated with αCD3 or αCD3/αCD28 for the time indicated. Full gating strategies from representative plots are shown in S1 Gating Strategy. PD-1, Programmed cell death protein 1; PD-L1, PD-1 ligand 1. (TIF) [file pbio.3001199.s007.tif]

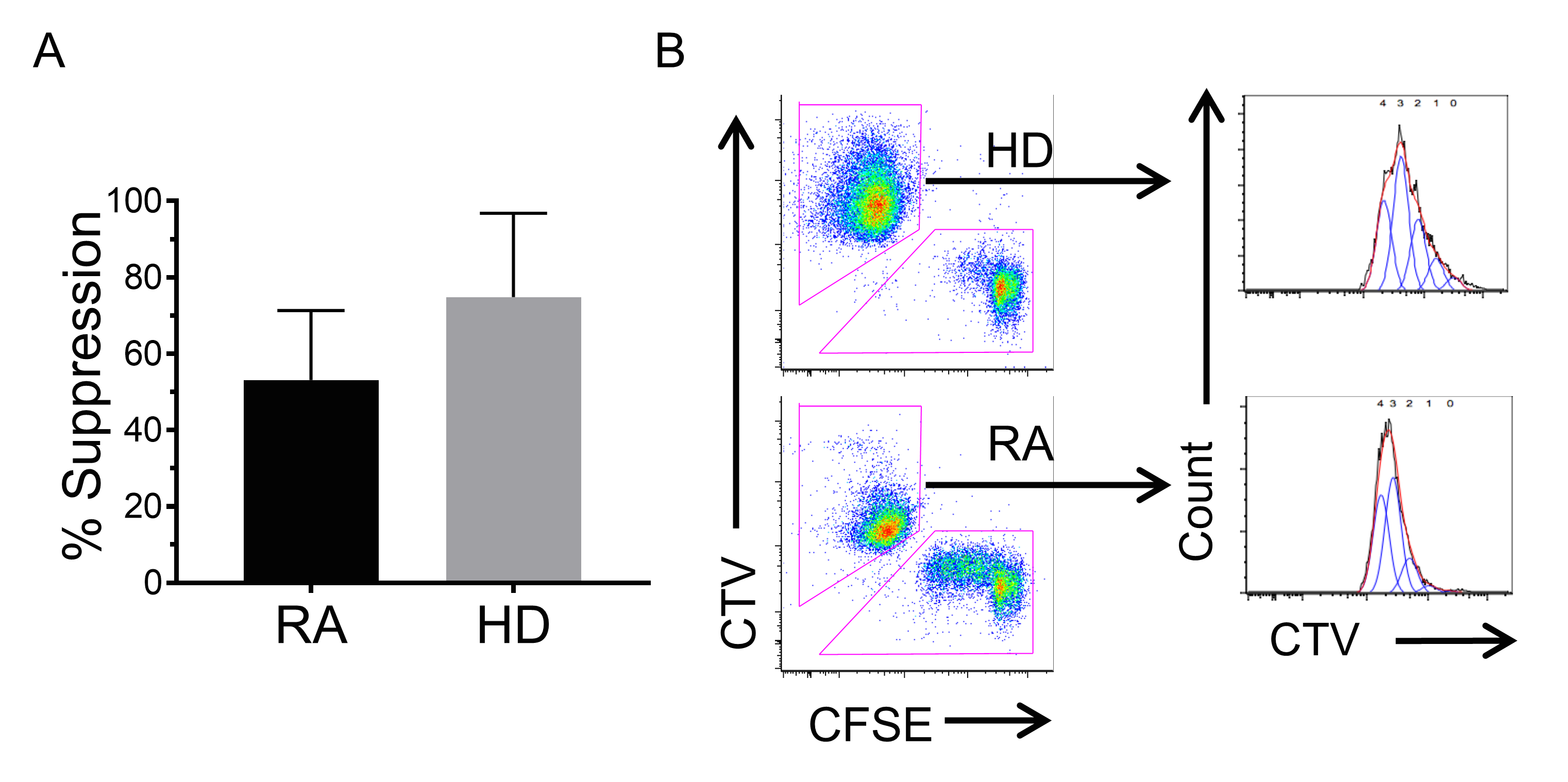

Supplement: S8 Fig — (A) Suppression of CD4+CD25+FOXP3+ T cells from HD and RA following PD-L1 engagement at 1:20 ratio. (B) Representative histograms showing CFSE dilution of effector CD4+ T cells (1 × 10^5^) activated with αCD3/αCD28 beads at 40:1 (cell/bead) ratio and cultured alone or in the presence of CTV-labeled CD4+CD25+FOXP3+ T cells from HD and RA following PD-L1 engagement cells for 5 days. Values for each data point can be found in S1 Data. Full gating strategies from representative plots are shown in S1 Gating Strategy. CFSE, Carboxyfluorescein succinimidyl ester; CTV, Cell Trace Violet; HD, healthy donor; PD-L1, PD-1 ligand 1; RA, rheumatoid arthritis. (TIF) [file pbio.3001199.s008.tif]
