# Supplementary material for: PD-L1 signaling on human memory CD4+ T cells induces a regulatory phenotype
Source: PLoS Biol. 2021 Apr 26;19(4):e3001199. doi: 10.1371/journal.pbio.3001199 (PMC8101994; doi:10.1371/journal.pbio.3001199)
Supplement: S1 Table — CCP, cyclic citrullinated peptides; csDMARDs, conventional synthetic Disease Modifying Anti-Rheumatic Drugs; n, number; RA, Rheumatoid Arthritis; RF, Rheumatoid Factor; SD, Standard Deviation. 1csDMARDs include Methotrexate, Sulfasalazine, Hydroxychloroquine, either alone or in combination. 2Biologics includes certolizumab-pegol (anti-TNFα). (DOCX) [file pbio.3001199.s009.docx]

| **Patients diagnosed with RA (n=16)** | | |
| --- | --- | --- |
| Age, years [mean±SD] | | 48.5±12.4 |
| Females [% (n)] | | 68.75% (11) |
| Anti-CCP+ [% (n)] | | 81.25% (13) |
| RF+ [% (n)] | | 68.75% (11) |
| Anti-CCP+ and/or RF+ [% (n)] | | 87.5% (14) |
| Treatment [% (n)] | None | 6.25% (1) |
|  | scDMARDs ^1^ | 93.75% (15) |
|  | Biologics ^2^ | 6.25% (1) |
| Concomitant Steroids [% (n)] | | 18.75% (3) |
