# Supplementary material for: PD-L1 signaling on human memory CD4+ T cells induces a regulatory phenotype
Source: PLoS Biol. 2021 Apr 26;19(4):e3001199. doi: 10.1371/journal.pbio.3001199 (PMC8101994; doi:10.1371/journal.pbio.3001199)
Supplement: S2 Table — List of antibodies used in this study including clones, fluorochromes, and suppliers. (DOCX) [file pbio.3001199.s010.docx]

| **Marker** | **Clone** | **Flurochrome** | **Supplier** | **Cat. No.** |
| --- | --- | --- | --- | --- |
| HLA-A2 | REA517 | PE-Vio770 | Miltenyi Biotec | 130-107-932 |
| CD4 | OKT4 | Brillant Violet 605 | BioLegend | 317438 |
| CD25 | BC96 | APC/Cy7 | BioLegend | 302614 |
| CD45RA | HI100 | Brillant Violet 785 | BioLegend | 304140 |
| CD45RO | UCHKL | FITC | BioLegend | 304242 |
| FOXP3 | PCH101 | FITC | eBioscience | 11-4776-42 |
| FOXP3 | 206D | Alexa Fluor 647 | BioLegend | 320114 |
| CTLA-4 | BNI3 | PE | BD Bioscience | 555853 |
| PD-L1 | MIH1 | PE-Cy7 | BD Bioscience | 558017 |
| PD-1 | EH12.2H7 | Brilliant Violet 421 | BioLegend | 329920 |
| pSTAT3 (pY705) | 4/P-STAT3 (pY705) | Alexa Fluor 488 | BD Bioscience | 557814 |
| pSTAT5 (pY694) | 47/Stat5(pY694) | Pacific Blue | BD Bioscience | 560311 |
| pERK1/2 (Thr202/ Tyr204) | 6B8B69 | PE | BioLegend | 369506 |
| pAKT (Ser473) | D9E | Alexa Fluor 647 | Cell Signaling Technology | 4075S |
