# Supplementary material for: PD-L1 signaling on human memory CD4+ T cells induces a regulatory phenotype
Source: PLoS Biol. 2021 Apr 26;19(4):e3001199. doi: 10.1371/journal.pbio.3001199 (PMC8101994; doi:10.1371/journal.pbio.3001199)
Supplement: S3 Table — List of antibodies used in this study including clones, metalTag, and suppliers. (DOCX) [file pbio.3001199.s011.docx]

| **Marker** | **Clone/reagent** | **Supplier** | **Cat.No.** | **Channel/**  **mass** |
| --- | --- | --- | --- | --- |
| CD45 | HI30 | Fluidigm | 3089003B | 89 |
| CCR6 | G034E3 | Fluidigm | 3141003A | 141 |
| CD62L | DREG-56 | BioLegend | 304835 | 142 |
| CD45RA | HI100 | Fluidigm | 3143006B | 143 |
| CD31 | WM59 | Fluidigm | 3145004B | 145 |
| CD8 | RPA-T8 | Fluidigm | 3146001B | 146 |
| CD95 | DX2 | BioLegend | 305631 | 147 |
| CD274/PD-L1 | 29E.2A3 | Fluidigm | 3148017B | 148 |
| CCR4 | 205410 | Fluidigm | 3149003A | 149 |
| CD161 | HP-3G10 | BioLegend | 339919 | 150 |
| CD103 | Ber-ACT8 | Fluidigm | 3151011B | 151 |
| CD69 | FN50 | BioLegend | 310939 | 152 |
| TIGIT | MBSA43 | Fluidigm | 3153019B | 153 |
| CD3 | UCHT1 | Fluidigm | 3154003B | 154 |
| CD279/PD-1 | EH12.2H7 | Fluidigm | 3155009B | 155 |
| Helios | 22F6 | BioLegend | 137202 | 156 |
| CD134/OX40 | ACT35 | Fluidigm | 3158012B | 158 |
| CCR7 | G043H7 | Fluidigm | 3159003A | 159 |
| CD39 | A1 | Fluidigm | 3160004B | 160 |
| CD152/CTLA-4 | 14D3 | Fluidigm | 3161004B | 161 |
| CD27 | L128 | Fluidigm | 3162009B | 162 |
| CXCR3 | G025H7 | Fluidigm | 3163004B | 163 |
| CD28 | CD28.2 | BioLegend | 302937 | 164 |
| CD127 | A019D5 | Fluidigm | 3165008B | 165 |
| CCR10 | 314305 | R&D Systems | MAB3478 | 166 |
| ICOS | DX29 | BD Bioscience | 557801 | 167 |
| Ki67 | Ki67 | Fluidigm | 3168001B | 168 |
| CD25 | 2A3 | Fluidigm | 3169003B | 169 |
| CD7 | CD7-6B7 | BioLegend | 343111 | 170 |
| FOXP3 | 259D | BioLegend | 320202 | 171 |
| FOXP3 | PCH101 | eBioscience | 14-4776-82 | 171 |
| CD38 | HIT2 | Fluidigm | 3172007B | 172 |
| CXCR4 | 12G5 | Fluidigm | 3173001B | 173 |
| HLA-DR | L243 | Fluidigm | 3174001B | 174 |
| CD4 | RPA-T4 | Fluidigm | 3176010B | 176 |
| DNA | Iridium | Fluidigm | 201192A | 191 |
| Live/dead | Cisplatin | Sigma | 479306 | 195 |
