# Supplementary material for: PD-L1 signaling on human memory CD4+ T cells induces a regulatory phenotype
Source: PLoS Biol. 2021 Apr 26;19(4):e3001199. doi: 10.1371/journal.pbio.3001199 (PMC8101994; doi:10.1371/journal.pbio.3001199)

iTreg conversion gating strategy (FOXP3 vs CD25 expression) used in the following figures: Figure 1C, 2B, 3C, 4A, 7A and Sup Figure 1B

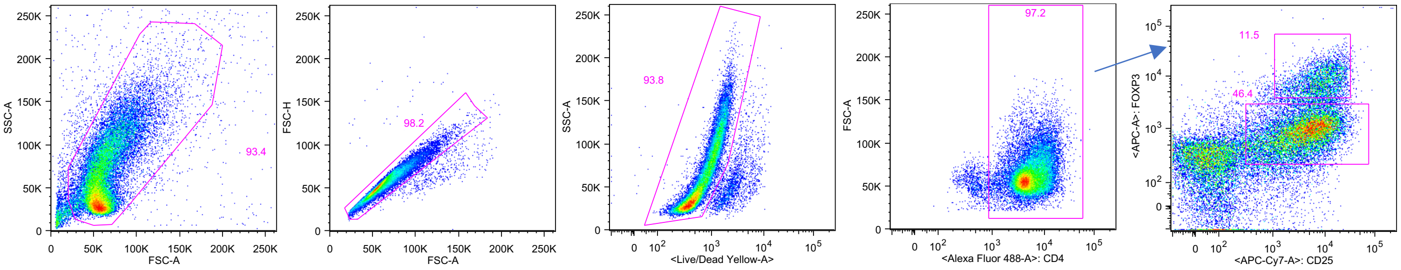

iTreg conversion gating strategy (CTLA-4 vs CD25 expression) used in the following figures: Sup Figure 1A and Sup Figure 1B (bottom panel)

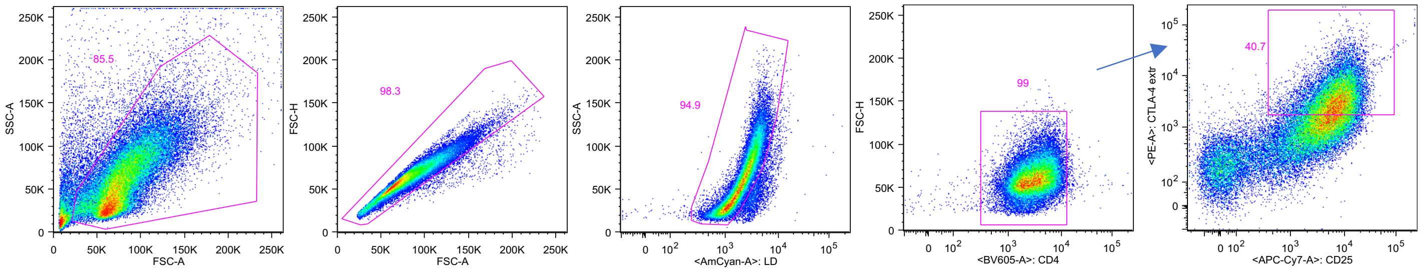

CTV gating strategy (FOXP3 vs CTV expression) used in Figure 2E

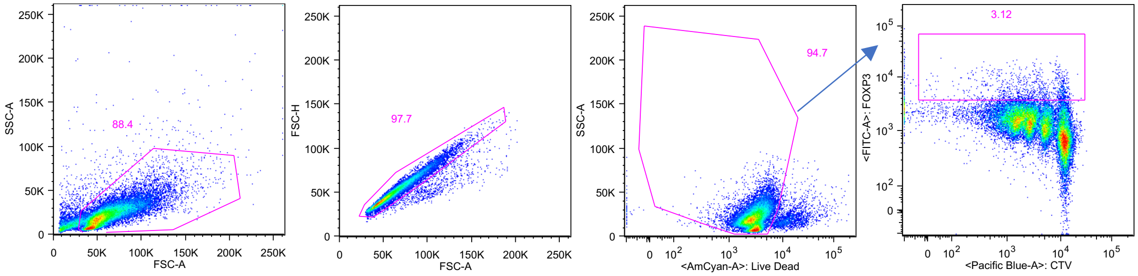

CRISPR/Cas9-*PD-1* gene editing efficiency (CD25 vs PD-1 expression) gating strategy used in Figure 2A

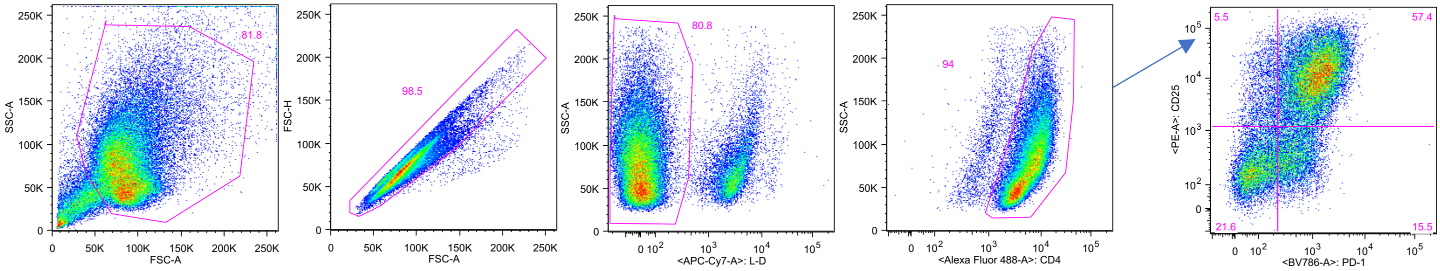

Suppression assay gating strategy used in the following figures: Figure 2F and 3D

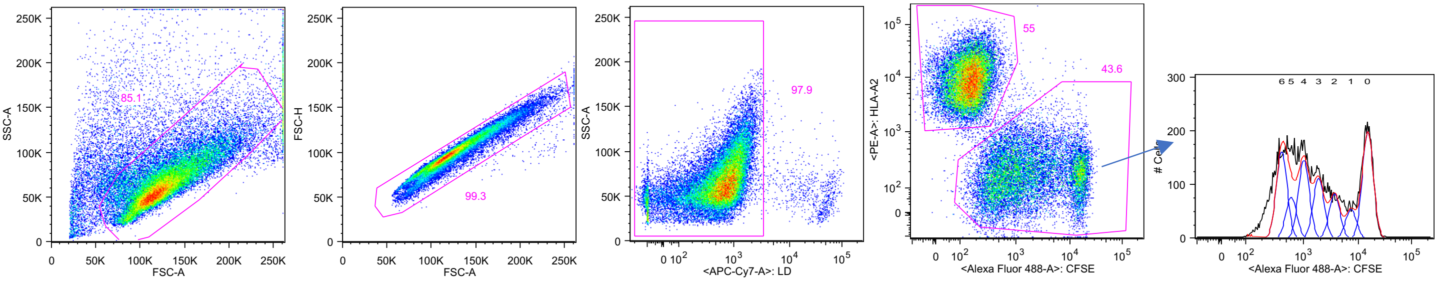

Phospho ERK ½ gating strategy used in the Figure 6A

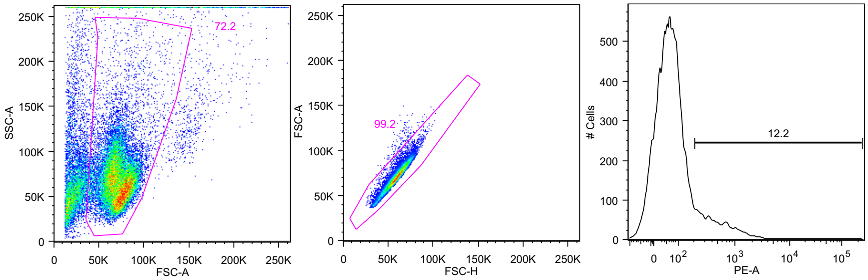

Phospho AKT, STAT3 and STAT5 gating strategy used in the following figures: Figures 5D and 6E

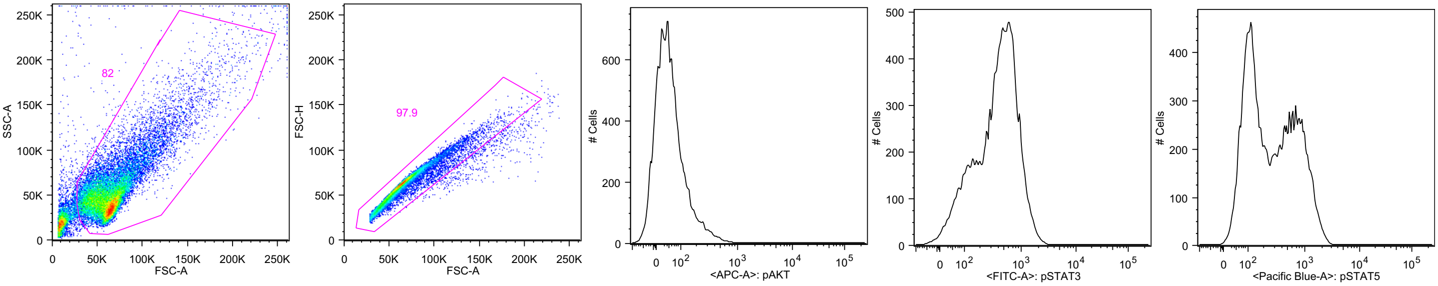

Supplement: S1 Gating Strategy — (PDF) [file pbio.3001199.s013.pdf]
