# Supplementary material for: PD-L1 signaling on human memory CD4+ T cells induces a regulatory phenotype
Source: PLoS Biol. 2021 Apr 26;19(4):e3001199. doi: 10.1371/journal.pbio.3001199 (PMC8101994; doi:10.1371/journal.pbio.3001199)
Supplement: S1 Raw Images — Blots are cut according to protein markers to allow analysis of multiple proteins from single cell lysates. “X” indicates irrelevant samples or irrelevant membranes. (PDF) [file pbio.3001199.s014.pdf]

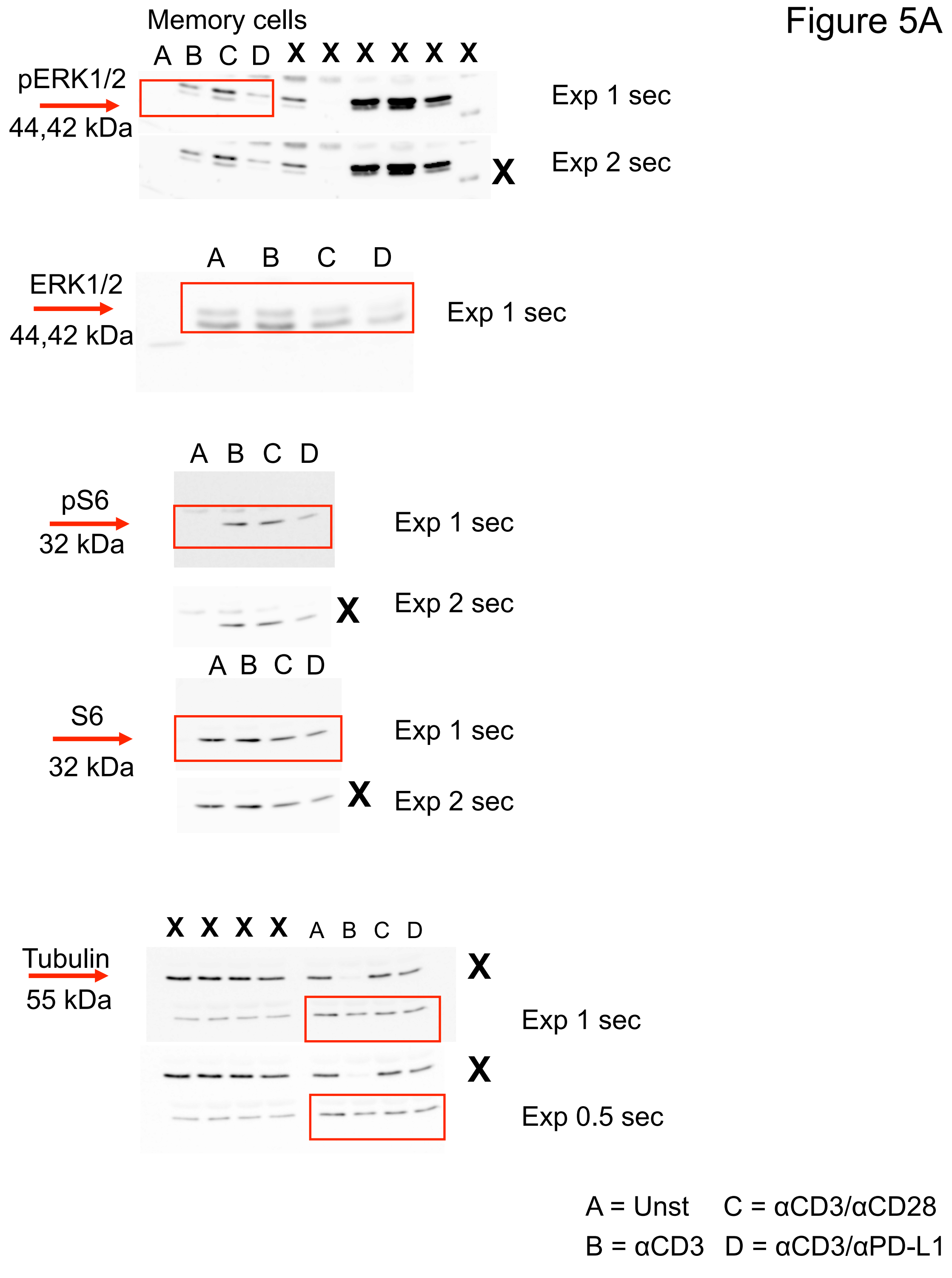
Memory cells
A B C D X X X X X X
pERK1/2
44,42 kDa
Exp 1 sec
Exp 2 sec
X
ERK1/2
44,42 kDa
A B C D
Exp 1 sec
pS6
32 kDa
A B C D
Exp 1 sec
Exp 2 sec
X
S6
32 kDa
A B C D
Exp 1 sec
Exp 2 sec
X
Tubulin
55 kDa
X X X X A B C D
X
Exp 1 sec
X
Exp 0.5 sec
A = Unst
C = αCD3/αCD28
B = αCD3
D = αCD3/αPD-L1

Figure 5A

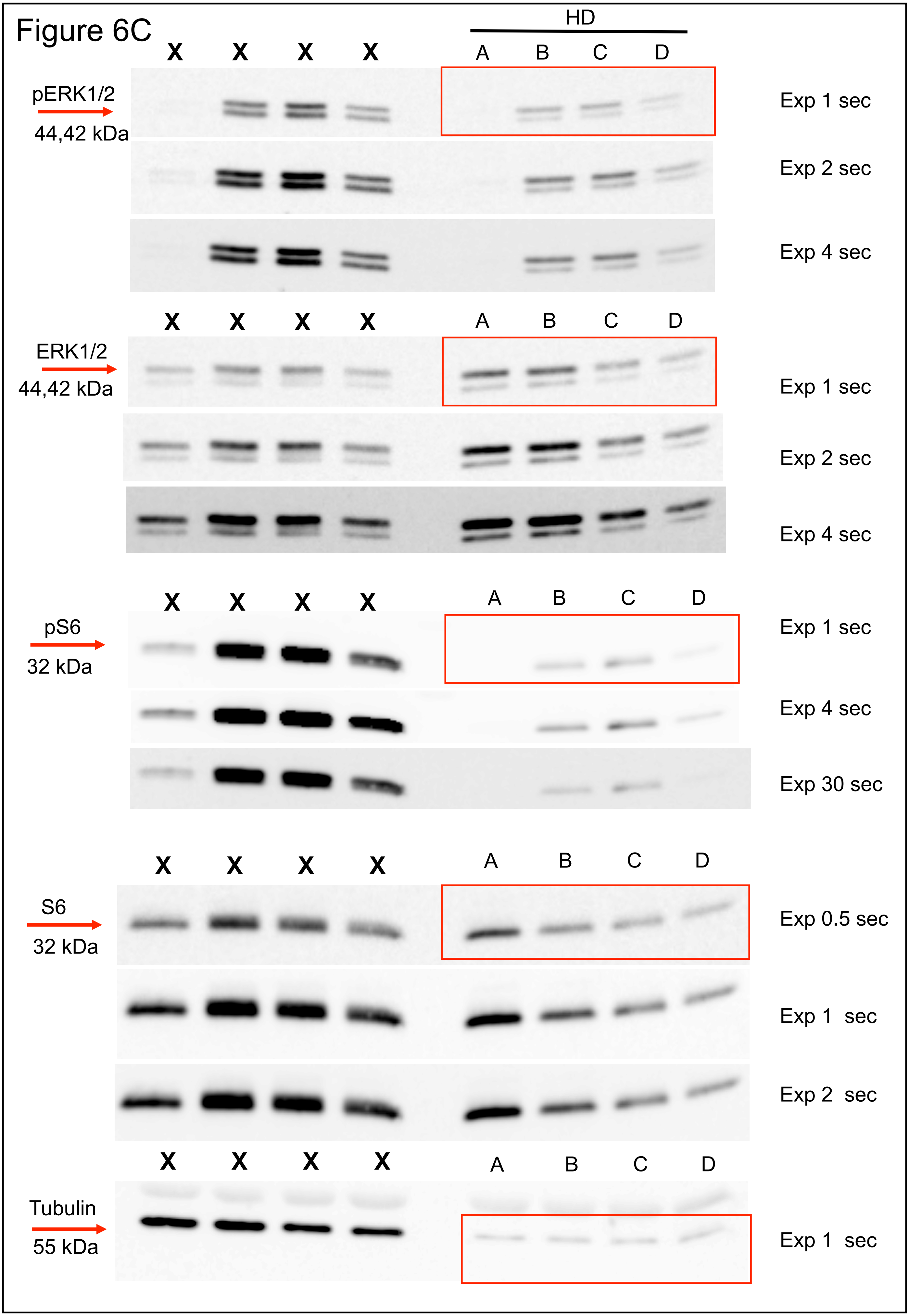

Figure 6C
HD
X X X X A B C D
pERK1/2
44,42 kDa
Exp 1 sec
Exp 2 sec
Exp 4 sec
X X X X A B C D
ERK1/2
44,42 kDa
Exp 1 sec
Exp 2 sec
Exp 4 sec
X X X X A B C D
pS6
32 kDa
Exp 1 sec
Exp 4 sec
Exp 30 sec
X X X X A B C D
S6
32 kDa
Exp 0.5 sec
Exp 1 sec
Exp 2 sec
X X X X A B C D
Tubulin
55 kDa
Exp 1 sec

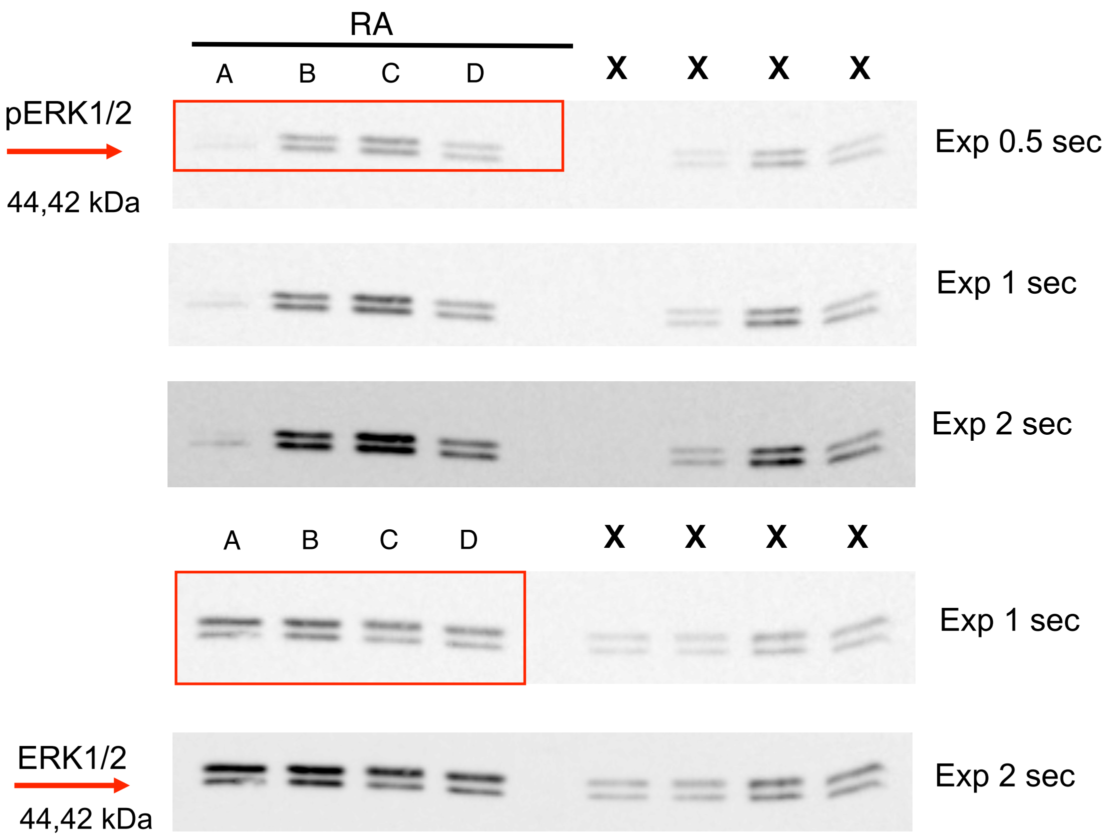

A = Unst C = αCD3/αCD28
B = αCD3 D = αCD3/αPD-L1

Figure 6D

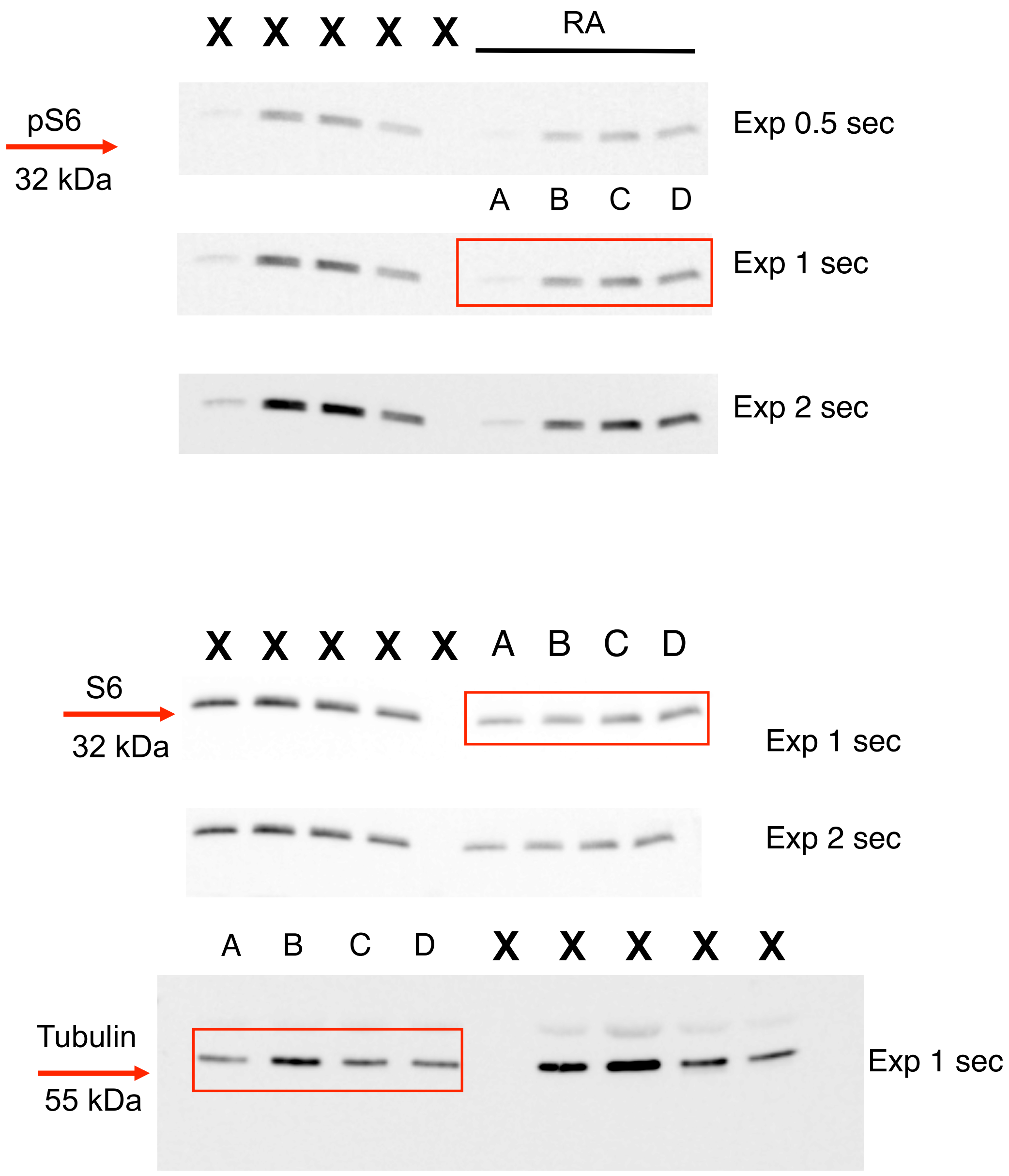

A = Unst   C = αCD3/αCD28

B = αCD3   D = αCD3/αPD-L1

Figure 6D
